# Effect of Dietary Approaches on Glycemic Control in Patients with Type 2 Diabetes: A Systematic Review with Network Meta-Analysis of Randomized Trials

**DOI:** 10.3390/nu15143156

**Published:** 2023-07-15

**Authors:** Tiantian Jing, Shunxing Zhang, Mayangzong Bai, Zhongwan Chen, Sihan Gao, Sisi Li, Jing Zhang

**Affiliations:** 1 School of Public Health, Shanghai Jiao Tong University School of Medicine, Shanghai 200025, China; jingtt312@shsmu.edu.cn (T.J.);; 2 Department of Global Public Health/Media, Culture, and Communication, Steinhardt School of Culture, Education, and Human Development, New York University, New York, NY 10016, USA; 3 School of Public Health, University of Washington Seattle Campus, Seattle, WA 98105, USA

**Keywords:** Type 2 diabetes, dietary patterns, network meta-analysis

## Abstract

Background: Dietary patterns play a critical role in diabetes management, while the best dietary pattern for Type 2 diabetes (T2DM) patients is still unclear. The aim of this network meta-analysis was to compare the impacts of various dietary approaches on the glycemic control of T2DM patients. Methods: Relevant studies were retrieved from PubMed, Embase, Web of Knowledge, Cochrane Central Register of Controlled Trials (CENTRAL), and other additional records (1949 to 31 July 2022). Eligible RCTs were those comparing different dietary approaches against each other or a control diet in individuals with T2DM for at least 6 months. We assessed the risk of bias of included studies with the Cochrane risk of bias tool and confidence of estimates with the Grading of Recommendations Assessment, Development, and Evaluation approach for network meta-analyses. In order to determine the pooled effect of each dietary approach relative to each other, we performed a network meta-analysis (NMA) for interventions for both HbA1c and fasting glucose, which enabled us to estimate the relative intervention effects by combing both direct and indirect trial evidence. Results: Forty-two RCTs comprising 4809 patients with T2DM were included in the NMA, comparing 10 dietary approaches (low-carbohydrate, moderate-carbohydrate, ketogenic, low-fat, high-protein, Mediterranean, Vegetarian/Vegan, low glycemic index, recommended, and control diets). In total, 83.3% of the studies were at a lower risk of bias or had some concerns. Findings of the NMA revealed that the ketogenic, low-carbohydrate, and low-fat diets were significantly effective in reducing HbA1c (viz., −0.73 (−1.19, −0.28), −0.69 (−1.32, −0.06), and −1.82 (−2.93, −0.71)), while moderate-carbohydrate, low glycemic index, Mediterranean, high-protein, and low-fat diets were significantly effective in reducing fasting glucose (viz., −1.30 (−1.92, −0.67), −1.26 (−2.26, −0.27), −0.95 (−1.51, −0.38), −0.89 (−1.60, −0.18) and −0.75 (−1.24, −0.27)) compared to a control diet. The clustered ranking plot for combined outcomes indicated the ketogenic, Mediterranean, moderate-carbohydrate, and low glycemic index diets had promising effects for controlling HbA1c and fasting glucose. The univariate meta-regressions showed that the mean reductions of HbA1c and fasting glucose were only significantly related to the mean weight change of the subjects. Conclusions: For glycemic control in T2DM patients, the ketogenic diet, Mediterranean diet, moderate-carbohydrate diet, and low glycemic index diet were effective options. Although this study found the ketogenic diet superior, further high-quality and long-term studies are needed to strengthen its credibility.

## 1. Introduction

Type 2 Diabetes (T2DM), characterized by hyperglycemia resulting directly from insulin resistance, and inadequate insulin secretion [1], has become a major threat to global public health [2]. In recent decades, large increases in T2DM prevalence have been demonstrated in virtually all regions of the world [3]. Growing concerns have been raised because an increase in T2DM prevalence will increase the number of chronic and acute diseases in the general population, with profound effects on quality of life, demand for health services, and economic costs [4].

In line with such a severe condition, medical nutrition therapy, through which diabetes and its consequences can be avoided or delayed, has garnered a high level of attention. Proper dietary patterns have been proven to have a vital role in preventing the progression of impaired fasting glucose (IFG) or impaired glucose tolerance (IGT) [5,6]. Previous studies have already found that dietary patterns play a critical role in T2DM management [7,8,9]. Thus, establishing the effect of dietary macronutrient approaches and popular named dietary programs is important. However, there is no evidence-based census demonstrating the best dietary pattern for T2DM patients [10]. Similarly, the American Diabetes Association (ADA) also indicates that there is no definitive evidence regarding the optimal dietary approach for the management of T2DM [7]. 

Currently, some pairwise meta-analyses have shown that dietary patterns, both dietary macronutrient and popular food-based dietary approaches, are effective in controlling glycemia, including HbA1c and fasting glucose [11,12,13]. However, others may show conflicting results for the effect of dietary approaches on markers of glycemia [14,15,16]. In addition, newly emerging dietary patterns are now being used in diabetes interventions, and their actual differences in controlling blood glucose are not clear [17,18]. There is some controversy surrounding the ketogenic diet, but recently, the ADA included the use of the ketogenic diet as a viable therapeutic option for the treatment of T2DM patients [19]. What is more troubling is that the comparative long-term effectiveness of dietary macronutrients and popular food-based dietary approaches for glycemic control for diabetes patients has not been examined [20]. So, there is a great need to compare the long-term (≥6 months) effect of multiple dietary approaches (that is, three or more) on glycemia, and define the promising dietary pattern, in order to provide direct recommendations to relevant patients.

To solve this question, a promising approach is network meta-analysis [21,22]. Compared to the current described pairwise meta-analysis, the methodology of network meta-analysis enables a simultaneous direct and indirect comparison of multiple interventions, forming a connected network, even when some comparisons have never been evaluated in a trial [23,24]. To the best of our knowledge, to date, network meta-analyses simultaneously comparing the effects of different dietary approaches on glycemic control of T2DM patients are still scarce. No published systematic review and meta-analysis has included the controversial ketogenic diet in comparison with other dietary patterns [25,26,27,28]. Therefore, the present systematic review and network meta-analysis aimed to include some newly emerging and controversial diets, determine the relative effectiveness and certainty of evidence among dietary macronutrient patterns and popular food-based dietary approaches on glycemic control (HbA1c, fasting glucose) in T2DM patients through the synthesis of available evidence from randomized trials.

## 2. Method

### 2.1. Registration

Our research protocol was registered in PROSPERO International Prospective Register of Systematic Reviews (https://www.crd.york.ac.uk/prospero/, accessed on 2 July 2022, identifier CRD42021264038). The present systematic review was planned, conducted, and reported according to the PRISMA guidelines and the corresponding extension for network meta-analyses [29,30].

### 2.2. Search Strategy

The literature searches were performed through PubMed, Embase, Web of Knowledge, and Cochrane Central Register of Controlled Trials (CENTRAL) (1949 to 31 July 2022) with no restriction to language and calendar date using a pre-defined search strategy (Supplementary File S1). In addition, we searched ISRCTN, ClinicalTrials.gov, and Clinicaltrials register.eu for unpublished trials or supplementary data for potentially eligible RCTs.

Moreover, the reference lists from the identified articles were screened to search for additional relevant studies. Searches were conducted by two authors with disagreements being resolved by the involvement of another reviewer. 

#### 2.2.1. Inclusion Criteria

1. Randomized controlled trials between different dietary approaches (energy-restricted diets, iso-caloric, or ad libitum diets):
  (1) Low-carbohydrate diet: less than 25% carbohydrate intake of total energy intake [31];
  (2) Moderate-carbohydrate diet: 25% to 45% carbohydrate intake of total energy intake [31];
  (3) Ketogenic diet: 5% to 10% carbohydrate intake of total energy intake, replacing the remaining with dietary fat and adequate protein (1 g/kg) [32];
  (4) Low-fat diet: less than 30% fat of total energy intake; high intake of cereals and grains; 10–15% protein intake [31];
  (5) High-protein diet: 25% to 35% protein intake of total energy intake [33];
  (6) Mediterranean diet: a daily abundance of vegetables, a variety of minimally processed whole grain bread, and other cereals and legumes as the staple food, nuts and seeds, fresh fruit as the typical daily dessert; sweets based on nuts, olive oil, and honey consumed only during celebratory occasions; cold pressed extra-virgin olive oil (EVOO), nuts and seeds as the principal source of fat; a low to moderate consumption of dairy products (mainly local cheese and yogurt) consumed in low amounts; a moderate consumption of fish, poultry, and eggs, a low consumption of red meat (once a week approximately), and a moderate consumption of wine, normally with meals [34];
  (7) Paleolithic diet: consumption of lean meat, fish, fruit, leafy and cruciferous vegetables, root vegetables, eggs, and nuts, while excluding dairy products, cereal grains, beans, refined fats, sugar, candy, soft drinks, beer, and extra addition of salt [35];
  (8) Nordic diet: consumption of traditional foods from the Nordic countries (the Scandinavian region), including whole grains, fruits (such as apples, pears, and berries), low-fat dairy products, fatty fish such as salmon, cabbage and root vegetables [18];
  (9) DASH (dietary approach to stop hypertension): high intake of fruits, vegetables, low-fat dairy products, and whole grains, and low in sodium [36];
  (10) Vegetarian/vegan diet: no meat and fish/ no animal products [37];
  (11) Low glycemic index diet (low GI/GL diet): consumption of food containing most carbohydrates from low-GI sources, such as beans, peas, lentils, pasta, pumpernickel bread, bulgur, parboiled rice, barley, and oats [11,38];
  (12) Portfolio dietary pattern: 1–3 g/day plant sterols (plant-sterol containing margarines, supplements), 15–25 g/day viscous fibers (gel-forming fibers, such as from oats, barley, psyllium, legumes, eggplants, okra), 35–50 g/day plant protein (such as from soy and pulses) and 25–50 g/day nuts (including tree nuts and peanuts [39];
  (13) Recommended diet (e.g., advice based on ADA guidelines) [40,41,42,43];
  (14) Control diet/usual diet (e.g., not changing usual diet) [25]: The control diet was used as our reference diet and presented results for the other diets against the reference diet.
  The classification of dietary approaches was derived from the original studies whenever possible. However, some dietary approaches can have important overlap with others in the macronutrient distribution. When a dietary approach could be classified as one of the specific dietary approaches (i.e., Mediterranean diet, Paleolithic diet), such classification was preferred over the classification based on macronutrient distribution of the diet. Meanwhile, if a dietary approach was initially claimed to be a low-carbohydrate diet, it would be priorly classified as a moderate-carbohydrate diet rather than a low-fat diet (based on the macronutrient classification in the original study) when it does not meet the criteria for a low-carbohydrate diet. For instance, a trial that was initially categorized as a low-carbohydrate diet [44] was reclassified as a moderate-carbohydrate diet in this study, based on the inclusion criteria for moderate-carbohydrate diets. Adjustments were made to ensure consistency with the classification standards used in our research.

2. Minimum intervention period of 6 months;
3. Participants with a mean age ≥ 18 years;
4. T2DM patients follow the diagnosis criteria of the ADA or according to internationally recognized standards [1].
5. The outcomes include at least one of HbA1c (%) and fasting glucose (mmol/L), as the main outcomes.

#### 2.2.2. Exclusion Criteria

1. Randomized trials including pregnant women, children, and adolescents, patients with abnormal glucose metabolism, chronic kidney disease, and disordered eating patterns;
2. Cross-over trials, single-arm trials, and study protocols; nonoriginal studies, including reviews, letters, case reports, or papers that did not provide accurate and clear data;
3. Intervention studies solely based on dietary supplements or single foods;
4. Intervention studies using dietary supplements as placebo;
5. Intervention studies using the medication as a placebo;
6. The same type of diet only changes one or a few of its components (e.g., a Mediterranean diet with avocados vs. a Mediterranean diet with nuts);
7. Interventions based on very low energy diets (i.e., <600 kcal/day);
8. Interventions claimed to be some kind of dietary pattern, but did not meet our criteria.

### 2.3. Data Extraction

The reviewers independently screened titles, abstracts, and full text, with any uncertainties regarding eligibility for inclusion resolved by discussion. All possibly relevant publications will be obtained in full if available, and reviewed for inclusion or exclusion by two independent reviewers. Data extraction will be carried out by one reviewer, with a second reviewer performing a quality check on a random sample (~10%). After the determination of the study selection, the following characteristics were extracted onto a Microsoft Excel spreadsheet (XP professional edition; Microsoft Corp., Redmond, WA, USA): the family name of the first author, year of publication, country, sample size, study duration, mean baseline age, % female, diabetes medication, description of the different dietary arms, energy restriction or not, in coordination or not (i.e., with exercise), drop-out rates and adverse events. Outcome data include post-intervention values with corresponding standard deviations for HbA1c and fasting glucose.

### 2.4. Risk of Bias Assessment

The revised Cochrane Risk of Bias Tool for Randomized Trials (RoB version 2.0) was used to assess the risk of bias (RoB) of the included RCTs [45]. Two reviewers independently assessed the risk of bias in the studies we finally select. The following domains of bias were detected: Randomization process, deviations from intended interventions, missing outcome data, measurement of the outcome, and selection of the reported result. Due to the inherent difficulty of implementing blinding in RCTs involving dietary patterns, we had taken this factor into consideration. If there were discrepancies, the discrepancies were resolved through discussion with a third team member until we reached a consensus. All studies were included in the synthesis regardless of the assessment of their quality if they contributed conceptually.

The overall risk of bias in each study was categorized as low risk of bias, some concerns, or high risk of bias.

### 2.5. Dealing with Missing Data

If the post-intervention values with the corresponding standard deviations were not available, the change scores with the corresponding standard deviations were used, according to the guidelines of the Cochrane Handbook [46]. When standard deviations were not available, we estimated them from standard errors, *p*-values, and confidence intervals.

### 2.6. Statistical Analysis

To compare the effects on glycemic control (changes in HbA1c and fasting glucose) between the dietary patterns, we used STATA version 16.0 (Stata Corp., College Station, TX, USA) (network package [47]) and produced presentation tools with the network graphs package [48]. Calculations were fitted in a frequentist framework. Direct comparisons between different dietary approaches were illustrated by using a network diagram [49], where the size of the nodes was proportional to the sample size of each dietary intervention and the thickness of the lines was proportional to the number of studies available. Heterogeneity was tested by Cochran’s Q test. *I*² of >50% was considered as substantial heterogeneity. Random-effects models were used to analyze the association between the dietary approaches and glycemia if *I*² > 50%, while fixed-effects models were applied if *I*² ≤ 50%. An indirect effect estimate was then calculated by comparing two interventions, and the control group was a common comparator. The outcomes were reported in terms of mean difference between the two interventions with a corresponding 95% credible interval (CI). The surface under the cumulative ranking curve (SUCRA) was used to estimate the ranking probabilities of the intervention effect. We constructed a cluster plot of SUCRA values for HbA1c and fasting glucose to assess both outcomes simultaneously.

As the networks that were studied included multiple closed loops, examinations for the inconsistency of direct and indirect evidence were carried out. To evaluate the inconsistency in the data, we performed the loop-specific approach, to detect loops of evidence that might present important inconsistency [50].

We produced comparison-adjusted funnel plots to explore publication bias or other small study effects, for all available comparisons [51]. Symmetry around the effect estimate line indicates an absence of publication bias or small study effects. The Egger’s asymmetry test was also performed for further confirmation [52].

### 2.7. Subgroup and Sensitivity Analyses

Subgroup analyses, according to the study duration (<12 months vs. ≥12 months), drop-outs (≤10% vs. >10%), and sample size (<100 vs. ≥100), were performed for HbA1c and fasting glucose. For sensitivity analysis, we analyzed the studies in which those with a higher risk of bias were not included. We ran a meta-regression analysis to investigate the association between the primary outcome (HbA1c and fasting glucose) and mean weight change, mean age, calorie restriction, co-intervention of exercise, and diabetes medications.

### 2.8. Credibility of the Evidence

The online tools of Confidence in Network Meta-Analysis (CINeMA) were used by a researcher to grade the quality of the evidence based on six domains: within-study bias, reporting bias, indirectness, imprecision, heterogeneity, and incoherence. Each domain is rated as having “major concerns”, “some concerns”, or “no concerns” [53].

## 3. Results

### 3.1. Search Results and Study Selection

As of 20 July 2022, a total of 8515 articles were identified in the initial literature search. One hundred and five studies were identified as potentially relevant after title and abstract screenings, of which 55 studies were further excluded after full text screening for reasons in Supplementary Table S1.

**Figure 1:**
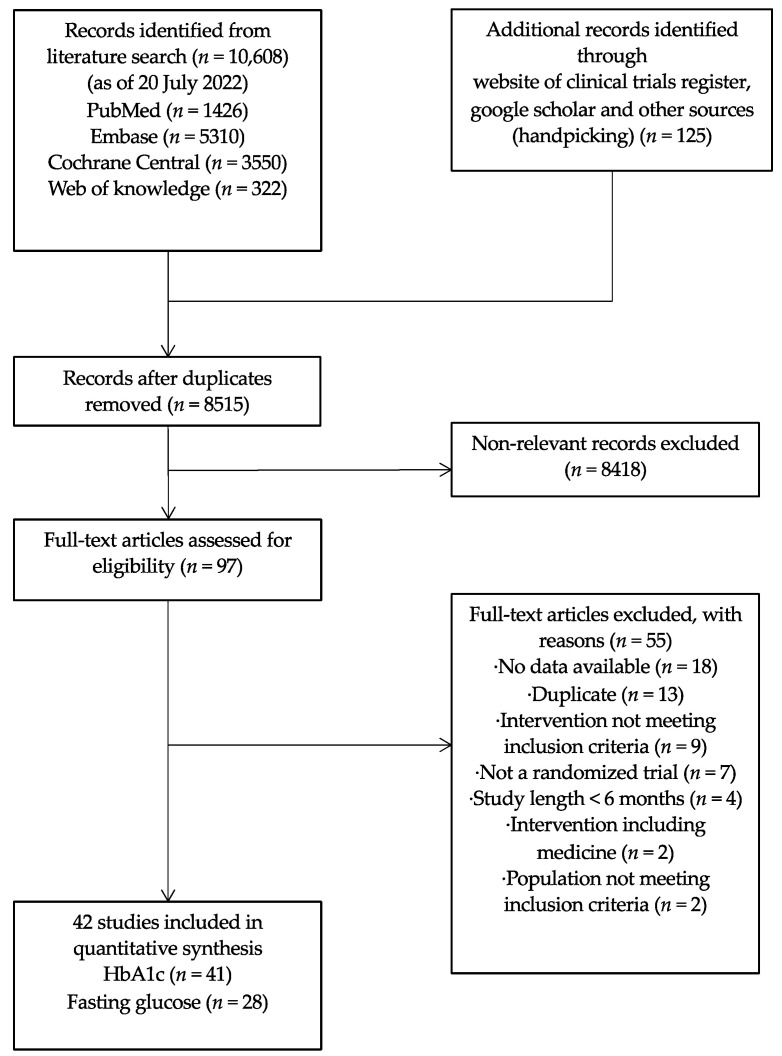
Flow diagram of study selection.

Forty-two studies (involving 4809 participants and conducted between 1993 and 2022) were finally included in the network meta-analysis [44,54,55,56,57,58,59,60,61,62,63,64,65,66,67,68,69,70,71,72,73,74,75,76,77,78,79,80,81,82,83,84,85,86,87,88,89,90,91,92,93,94] (Figure 1). We meant to compare the effects of 14 dietary patterns on the controlling of diabetes, as our searching strategies showed, yet 10 dietary patterns were finally included for a lack of such studies meeting our eligibility criteria of the other 4 dietary patterns (portfolio diet, Nordic diet, Paleolithic diet, and DASH).

### 3.2. Study Characteristics

Eleven trials were conducted in North America, 11 in Europe, 11 in Asia, and 8 in Australia and New Zealand. Study durations ranged from 6 months to more than 3 years. In terms of subjects, trials primarily included overweight and obese patients with diabetes, with 15/42 (35.7%) trials including participants using insulin. Reported patients’ average ages ranged between 42.5 and 67.4, and female proportions ranged between 10.4% and 79.7%. Drop-outs were commonly reported, with 32/42 (76.2%) studies reporting missing data. In terms of the implementation of the intervention, 31/42 (73.8%) studies were implemented by dietitians or nutritionists, while 3/42 (7.14%) were implemented by doctors or nurses training in clinical psychology or diabetes education; eight studies did not report relevant details. Thirty-seven included studies were two-arm trials, and five were three-arm trials. Thirteen had a study duration ranging from 6 to 12 months, while the other twenty-nine studies were conducted for at least 12 months. The general and specific study characteristics are summarized in Table 1 and Supplementary Table S2.

In terms of outcome, 41 RCTs evaluated HbA1c (%; *n*_patients_ = 4721) [44,54,55,56,57,58,59,60,61,62,63,64,65,66,67,68,69,70,71,72,73,74,75,76,78,79,80,81,82,83,84,85,86,87,88,89,90,91,92,93,94] while 28 RCTs evaluated fasting glucose (mmol/L; *n*_patients_ = 3360) [44,54,56,58,60,61,63,65,66,67,68,70,71,72,75,77,78,79,80,81,82,83,85,87,89,90,93,94]. The most commonly used intervention was a low-fat diet [44,54,55,56,59,61,62,63,64,65,67,68,69,70,73,74,75,77,78,79,80,82,83,84,85,89,90,92], followed by a low-carbohydrate diet [62,68,71,73,74,79,80,84,86,88,92,94], while the least commonly used intervention was a Vegetarian/Vegan diet [60,72]. 

It is noteworthy that the definition of diets was heterogeneous in the included RCTs in aspects of (a) the delivery approach (viz., group meeting vs. dietary counseling), (b) the prescribed diet (viz., ad libitum, isocaloric, hypocaloric), and (c) in coordination or not (i.e., with exercise). As such interventions were balanced among groups, we still included these studies, and the information mentioned above is summarized. But the single trials were harmonized. Meanwhile, RCTs involving a control diet were also heterogeneous in terms of whether some dietary instruction was provided in the control condition. Therefore, it was decided before data analysis that these RCTs were further categorized into (i) recommended diets, in which a specific diet was recommended to patients in the control group (e.g., advice based on ADA guideline; 10 RCTs [57,60,66,71,72,76,86,88,94]), and (ii) for the control diet, no specific instruction was provided to patients (e.g., not changing usual diet; 13 RCTs [44,54,55,64,65,67,69,76,81,82,87,91,93]). This distinction further allows the comparison of intervention effects against different control conditions. 

Overall, recommended nutritional patterns were, in general, similar across all RCTs. To be specific, most of the recommended diets follow ADA guidelines; the details of the recommended diets we included are summarized in Supplementary Table S3. Considering the recommended diet did not strictly formulate macronutrient intake (e.g., the balance of the calories was covered by fat) [86], and additionally suggested sources of healthy food (e.g., suggest 50–60 E% carbohydrates mainly from fruit, vegetables, and whole-grain sources) [94], the recommended diet was therefore categorized as one of the reference groups.

### 3.3. Risk of Bias in Included Studies

**Figure 2:**
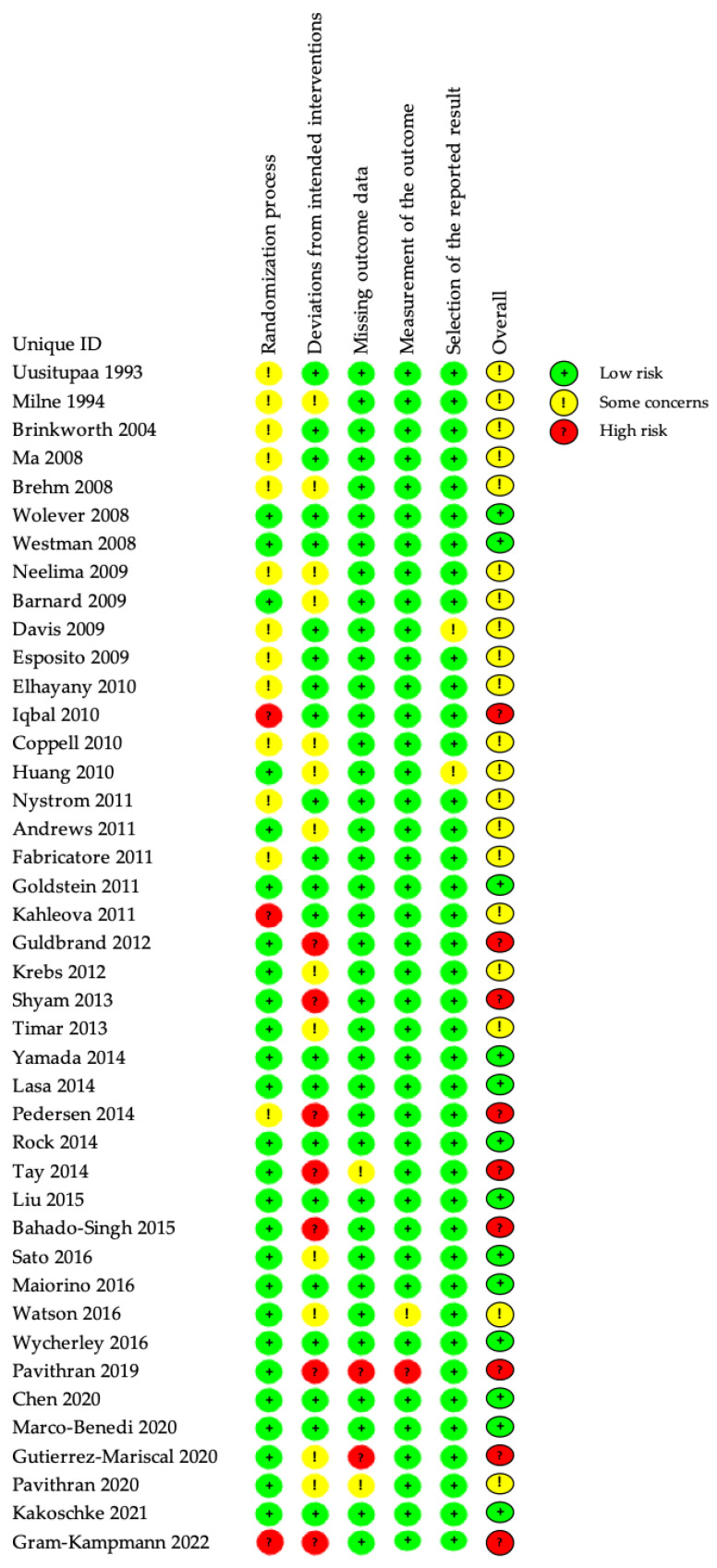
Risk of bias summary: Review authors’ judgements about each risk of bias item for each included study. Data from references [44,54,55,56,57,58,59,60,61,62,63,64,65,66,67,68,69,70,71,72,73,74,75,76,77,78,79,80,81,82,83,84,85,86,87,88,89,90,91,92,93,94].

**Figure 3:**
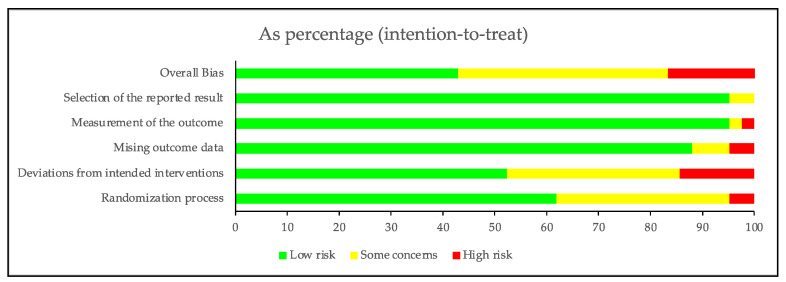
Risk of bias graph: Review authors’ judgements about each risk of bias item presented as percentages across all included studies.

All included studies were assessed by two authors independently and simultaneously. The results of the RoB analysis are summarized in Figure 2 and Figure 3. The overall risk of bias was rated as high for 7 (16.7%) studies and low for 18 (42.9%) studies. Among the five types of risk assessed, namely randomization process, deviations from intended intervention(s), missing outcome data, outcome measurement, and selection of the reported result, the first two types were the major risks of bias in the included studies. Studies were deemed somehow risky in the randomization process if the report of which lacked details or was indicative of failing double-blindness. Deviation from the intended intervention was considered mainly for the blinding of assessors or analysts and the potential deviation because of experimental contexts. Concerning the missing outcome data domain, most studies probably had data for all of, or nearly all of, the randomized participants, while for those that were considered some concern or high risk of bias, either important percentages of subjects that dropped out were found, or no information about missing data was reported. We have considered a drop-out rate of ≤10% as one of our criteria. Outcome measurement was considered lower in risk for most of the studies, as it was conducted by third-parties other than the researchers, except for one study [87], in which one resulted in limited information and the other did not describe the measurement. And two studies [73,87] were deemed medium risky in the selection of the reported result because of the absence of a prespecified trial protocol, so we assessed this domain as “some concerns”. Finally, it was noteworthy that the overall risk of bias was adjusted for studies only deemed risky in blinding [60,67,69,74,75,76,86], as the dietary intervention was naturally difficult to keep blinded to both patients and care-givers.

### 3.4. Effects of the Interventions

#### 3.4.1. Network Meta-Analysis of the Association between Dietary Patterns and the Glycemic Control

**Figure 4:**
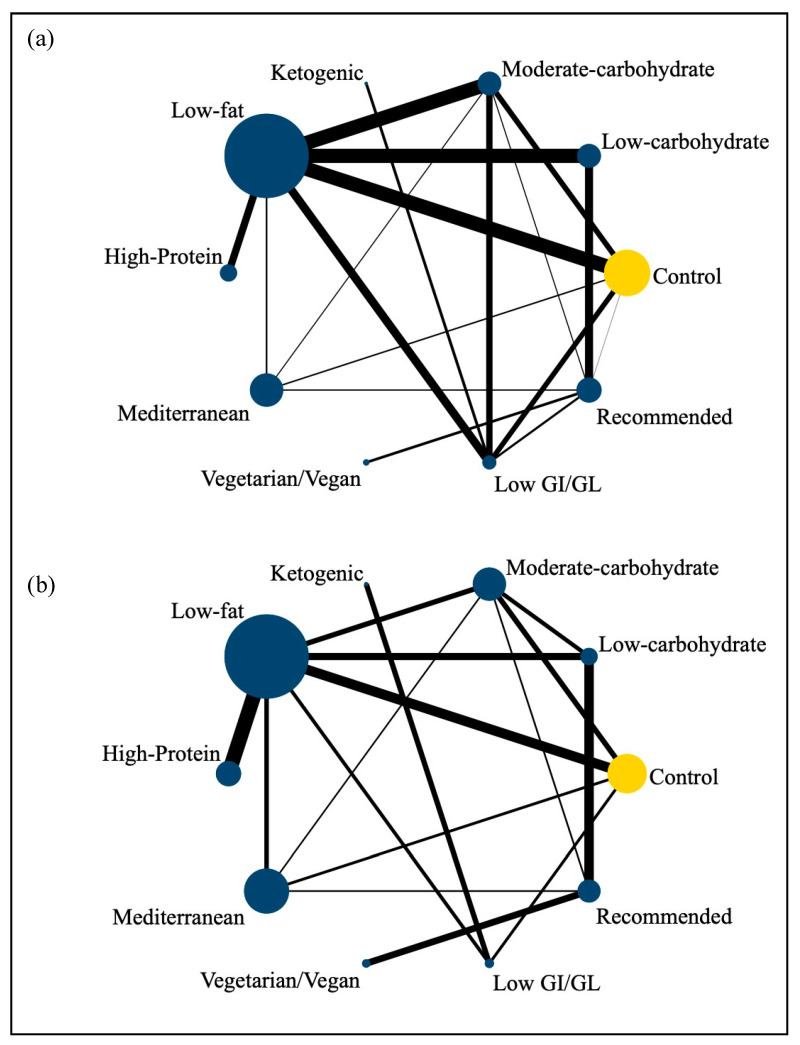
Network diagrams for direct comparison for HbA1c (panel (**a**)) and fasting glucose (panel (**b**)). The size of the nodes was proportional to the sample size of each dietary intervention and the thickness of the lines was proportional to the number of studies available.

Figure 4 shows the network diagrams of direct comparison for HbA1c (panel (a)) and fasting glucose (panel (b)). Nodes represent RCT conditions with their size reflecting the number of patients involved. Lines represent the RCTs comparing the conditions (nodes) connected with its widths reflecting the numbers of RCTs. For HbA1c, RCTs involving low-fat diets, compared to low-carbohydrate diets, dominated (*n* = 8); while for fasting glucose, RCTs involving low-fat diets, compared to high-protein diets, dominated (*n* = 5).

Table 2 summarizes the estimated effect size differences (MDs with 95% Cis) comparing every possible combination of two intervention approaches; results for HbA1c are presented below the diagonal, while those for fasting glucose are presented above the diagonal. For HbA1c (%), a greater reduction was found for ketogenic diets, low-carbohydrate diets, and low-fat diets than control diets (viz., −0.73 (−1.19, −0.28), −0.69 (−1.32, −0.06), and −1.82 (−2.93, −0.71)). In addition, a greater reduction of HbA1c was found in ketogenic diets than recommended diets (−1.33 (−2.48, −0.19)), high-protein diets (−1.40 (−2.62, −0.17)), low-carbohydrate diets (−1.49 (−2.71, −0.27)), and low-fat diets (−1.45 (−2.66, −0.25)). For fasting glucose (mmol/L), a greater reduction was found for moderate-carbohydrate diets, low glycemic index diets, Mediterranean diets, high-protein diets, and low-fat diets than for the control diets (viz., −1.30 (−1.92, −0.67), −1.26 (−2.26, −0.27), −0.95 (−1.51, −0.38), −0.89 (−1.60, −0.18) and −0.75 (−1.24, −0.27)). Additionally, a greater reduction was found for moderate-carbohydrate diets than recommended diets (−1.04 (−1.81, −0.28)), while no statistical difference was found for other comparisons of intervention approaches.

#### 3.4.2. SUCRA

**Figure 5:**
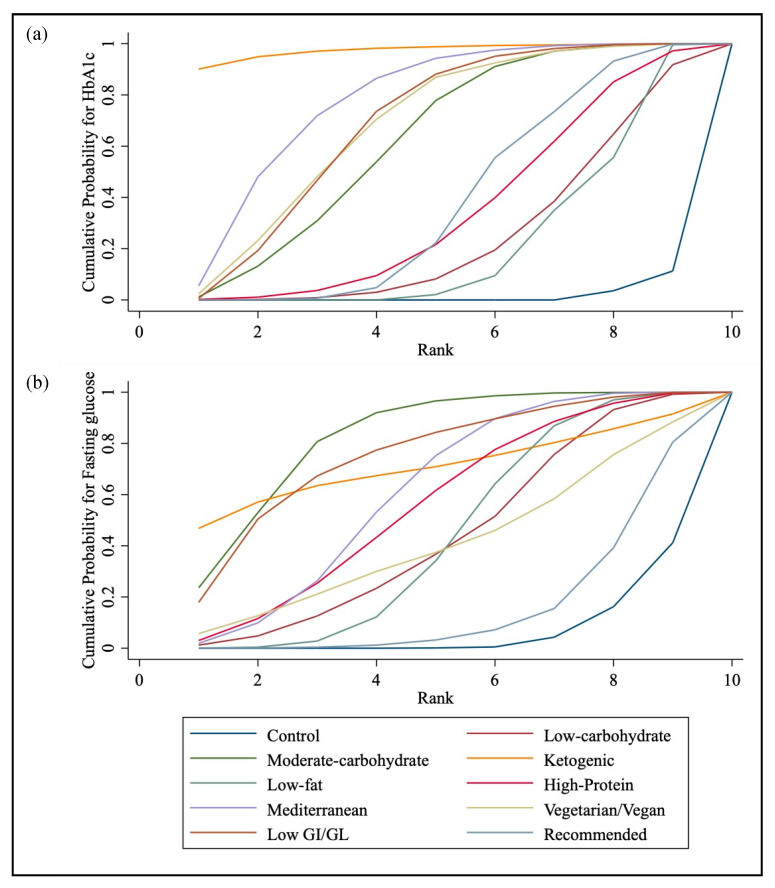
Panel (**a**): SUCRA for HbA1c, panel (**b**): SUCRA for fasting glucose.

The SUCRA values and ranks for each outcome (HbA1c and fasting glucose) were summarized in Table 3 and Figure 5. The top-three effective interventions were the ketogenic diets (97.5%), Mediterranean diets (78.1%), and low glycemic index diets (69%) for HbA1c, and moderate-carbohydrate diets (82.7%), low glycemic index diets (75.4%), and ketogenic diets (71%) for fasting glucose, respectively. 

**Figure 6:**
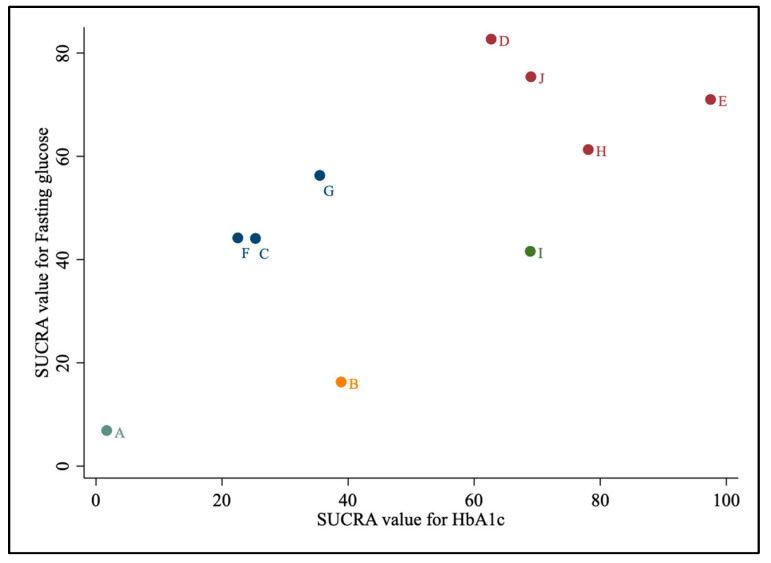
Clustered ranking plot. The clustered ranking plot according to the SUCRA values of HbA1c (%) and fasting glucose (mmol/L). The sum of the SUCRA values was derived from the mean ranking of effectiveness in a network. The dietary pattern in the upper-right quadrant represents the most effectiveness in glycemic control. A = Control diet, B = Recommended diet, C = Low-carbohydrate diet, D = Moderate-carbohydrate diet, E = Ketogenic diet, F = Low-fat diet, G = High-protein diet, H = Mediterranean diet, I = Vegetarian/Vegan diet, J = Low GI/GL diet.

Figure 6 shows the two-dimensional cluster plots that combine the SUCRA ranking for two outcomes. The same color represents the clusters with similar efficacy for the combination of both outcomes. The cluster of treatments on the right upper corner group ranked highest for both outcomes, while treatments on the left lower corner group ranked lowest. In particular, the clustered ranking plot for combined outcomes indicated the ketogenic, Mediterranean, moderate-carbohydrate, low GI/GL diets had promising effects for controlling HbA1c and fasting glucose. On the contrary, control diets showed a lower efficacy in both the two outcomes.

### 3.5. Inconsistency

The loop-specific approach was adopted for probing inconsistency. Inconsistency was found for HbA1c for comparisons between (a) low-carbohydrate and low-fat diets, (b) low-carbohydrate and recommended diets, and (c) the Mediterranean and recommended diets using the side-splitting approach. No significant inconsistency was found for fasting glucose. (Supplementary Tables S4 and S5).

### 3.6. Subgroup and Sensitivity Analyses

Sensitivity analyses concerning sample sizes, study durations, and drop-out rates were conducted, and the results are shown in Supplementary Tables S6–S19. We found that the moderate carbohydrate diet had a significant effect in reducing both HbA1c and fasting glucose in the long term (study duration ≥ 12 months), exhibited a bigger sample size (sample size ≥ 100), and resulted in a smaller proportion of drop-outs (≤10%), while the Mediterranean diet was found to be effective in the bigger sample size (sample size ≥ 100) and a smaller proportion of drop-outs (≤10%) for both outcomes as well. The ketogenic diet remained the most effective in reducing HbA1c in a smaller sample size (sample size < 100), while such an effect did not exist in fasting glucose. In the exploration of excluding studies of a higher risk of bias [68,78,81,87,89,94], we found that the effectiveness of the ketogenic diet and moderate carbohydrate diet on HbA1c and fasting glucose remained, respectively. Regarding fasting glucose, excluding studies with a higher risk of bias generally confirmed the results of the main outcome, while the effects of the moderate carbohydrate, Mediterranean, and high-protein diets were stronger compared to the control diet. Similar effects also existed in the sensitivity analysis of HbA1c. However, we also found that the long- and short-term effects of some dietary patterns were not sequenced similarly, such as the Mediterranean diet.

### 3.7. Small Study Effects and Publication Bias

The comparison-adjusted funnel plots for HbA1c suggested the possibility of publication bias or a small-sample effect. While the comparison-adjusted funnel plots for fasting glucose showed no significant asymmetric trend. (Supplementary Figures S7 and S8) In our study, the *p*-value of the Egger test yielded non-statistically significant findings for all of the outcomes of interest (*p*s > 0.164) when it was used to examine the presence of publication bias.

### 3.8. Meta-Regression and Additional Analyses

In univariate meta-regressions (i.e., mean weight change, mean age, calorie restriction, co-intervention of exercise, and diabetes medications), we found that the mean reductions of HbA1c and fasting glucose were only significantly related to the mean weight change of the subjects (Supplementary Figures S5 and S6), thereby showing that weight loss is a major contributing factor for glycemic control. 

For further exploration of weight change, we found that all dietary approaches were more effective in reducing body weight than recommended and control diets, with changes ranging between −6.26 and −2.06 kg. And the ketogenic and Mediterranean diets were the top-two effective interventions with relatively close SUCRA values (74.6% and 74.3%, respectively) (Supplementary Tables S22 and S23).

### 3.9. Adverse Events

Four (9.5%) studies reported adverse events during the intervention period, including headache, constipation, diarrhea, insomnia, back pain, fibrillation, and pneumonia [58,59,63,94]. In general, original authors deemed these adverse events unrelated to the study interventions or did not differ significantly between the groups, except for an increased frequency of gastrointestinal complaints in the low-carbohydrate group, reported by one study [94]. 

### 3.10. Credibility of the Evidence

Levels of evidence credibility were assessed via the CINeMA tool [53], following the approach suggested by Salanti et al. [95]. Surprisingly, the credibility of evidence was rated as very low to moderate for most comparisons regarding HbA1c and fasting glucose. An exception was that the credibility of evidence was high for the moderate-carbohydrate diets with the control diets regarding fasting glucose. Certainty of evidence judgments was mainly driven by major concerns regarding incoherence or imprecision for several comparisons (Supplementary Tables S20 and S21).

## 4. Discussion

Our network meta-analysis included 42 RCTs to assess the impact of dietary patterns on glycemic control in T2DM patients. By applying NMA, we ranked 10 dietary patterns (control diet, low-carbohydrate diet, moderate-carbohydrate diet, ketogenic diet, low-fat diet, high-protein diet, Mediterranean diet, Vegetarian/Vegan diet, low GI/GL diet, recommended diet), regarding their comparative efficacy for glycemic control in patients with T2DM. In this systematic review and NMA, ketogenic, low-carbohydrate, moderate-carbohydrate, low GI/GL, Mediterranean, high-protein, and low-fat diets significantly reduced HbA1c and fasting glucose compared to control diets. The clustered ranking plot for combined outcomes indicated the ketogenic, Mediterranean, moderate-carbohydrate, and low GI/GL diets had significant effects in controlling glycemia, while control diets showed the lowest efficacy. This indicates that interventions using dietary approaches are crucial for glycemic control, whereas continuing one’s usual diet was the worst option. Therefore, due to the positive trends in individual studies and our synthesis outcome in support of dietary approach interventions, the results may help the application of clinical practice. However, for most comparisons, the credibility of evidence was rated between very low and moderate. There remains insufficient evidence to definitively identify the optimal dietary approach.

In line with previous studies, the findings from this study indicate that dietary patterns reduce HbA1c, with the ketogenic, Mediterranean, and low GI/GL diets having promising effects. Zhou et al. found that ketogenic diet was an effective dietary intervention for body weight and glycemic control, as well as improved lipid profiles in overweight patients with T2DM [96]. The low GI/GL diet was found to have a clinically useful effect on medium-term glycemic control in patients with diabetes [11,97]. The meta-analysis conducted by Huo et al. found that a Mediterranean diet resulted in more significant improvements in glycemic control and weight loss compared to control diets [98]. 

Concerning mechanisms of action, on the one hand, carbohydrates are by far the most significant dietary contributor to elevated blood glucose, and restricting dietary carbohydrates can lower blood glucose levels. Diet can influence glycemic control by reducing the quantity and improving the quality of carbohydrate intake [27]. Consuming food items that are rich in high-glycemic carbohydrates can lead to rapid and significant increases in blood glucose and insulin levels, particularly in individuals with T2DM. Regular consumption of such foods can worsen hyperinsulinemia and amplify the associated atherogenic response [27,99]. For the ketogenic diet, it may also be related to the production of ketone bodies, which replace glucose as an energy source. This shift in energy source can contribute to appetite suppression and various improvements in metabolic markers (i.e., leptin, adiponectin, lipoproteins, lipogenesis, and insulin) [100]. While the Mediterranean and low GI/GL diets shared some common elements of a healthy diet, such as increasing the intake of monounsaturated fatty acids, polyunsaturated fatty acids, dietary fiber, and selecting to take medium-GI food [59,61], refined carbohydrates are reduced and whole grains are encouraged. So, regardless of the type of intervention, each diet encourages specific healthy elements supporting the current dietary recommendations, which is helpful to control glycemia [101,102]. On the other hand, obesity is highly prevalent in patients with T2DM, which is found to be associated with chronic inflammation statuses [103]. In a dose–response meta-analysis, each kg of mean weight loss was related to a mean HbA1c reduction of 0.1 percentage points, showing that glycemic control was strongly correlated with weight change [15,104]. In line with these findings, our meta-regression analysis showed the association between mean differences in HbA1c (%), fasting glucose (mmol/L), and mean weight reduction of patients between dietary approaches. However, there is still a lack of long-term, high-quality evidence on dietary patterns. 

Although the effects of dietary approaches on T2DM management are currently a hot topic, there remains a scarcity of studies examining the long-term effects of dietary approaches, specifically for glycemic control in T2DM patients [20]. And there are mixed views on the ketogenic diet, which has caused intense debate in both the scientific community and the general public [105]. It is worth noting that a long-term follow up is necessary for the study of dietary patterns to determine whether there are potential risks associated with the diet and to monitor the extent of those risks. Adherence is another critical point and is also emphasized in our study. 

First, previous studies have indicated that the impact of dietary interventions tends to diminish over time [25,27]. Based on previous research, we have set a cutoff point of 6 months as one of our inclusion criteria for long-term studies [106]. In contrast to previous studies that attempted to confirm short-term effects [107], our study included evidence of sustained intervention for at least six months, further confirming the effects of the dietary approaches for glycemic control. The stability of the results was also corroborated by sensitivity analysis.

Second, we extracted adverse events and drop-out rates of all studies to examine the adherence to interventions. 76.2% of the studies reported drop-outs, ranging from 2.2% to 52.8%. We took the drop-out rates as one of the criteria for assessing RoB. Four studies reported adverse events during the intervention period, in which one study reported an increased frequency of gastrointestinal complaints in the low-carbohydrate group. However, the targets were free-living population, so variability in adherence is likely. Self-reported dietary data have well-recognized limitations in accuracy, which are characterized by substantial underreporting and misreporting among overweight and obese individuals [44]. Focusing on and enhancing patients’ compliance with dietary intervention is crucial to ensure the practical significance of research findings. This aspect of real-world research deserves special attention.

Although no present studies met our inclusion criteria, Alireza et al. found that a Nordic diet might improve serum insulin and HOMA-IR levels [18]. Similarly, Eric et al. demonstrated the Paleolithic diet resulted in greater short-term improvements in metabolic syndrome components (waist circumference, triglycerides, HDL cholesterol, blood pressure (systolic and diastolic), and fasting glucose) than guideline-based control diets [108]. Future studies should further explore the long-term effects of dietary patterns (portfolio diet, Nordic diet, Paleolithic diet, and DASH) for their potential but promising effects on glycemic control.

Indeed, the Mediterranean diet is widely recognized for its health benefits [98], while the ketogenic diet has garnered significant attention for its potential effectiveness [17]. For instance, the Italian Society of Endocrinology has recommended a 12-week ketogenic diet treatment as part of a multidisciplinary weight management strategy for obese patients who have a clinically assessed need to lose weight rapidly [109]. Considering the challenges of adhering to a highly restrictive dietary regimen over a long-term period, researchers carried out a combination of biphasic ketogenic Mediterranean diet and Mediterranean diet protocol [110]. Over the 12-month study period, improvements in metabolic parameters, including glycemia levels, were observed. Combining interventions from different beneficial dietary approaches will become a growing trend to achieve long-term dietary management goals while ensuring effectiveness. Our findings provide evidence to support that the ketogenic diet can be one of the valuable options. 

As emphasized in the guidelines for T2DM management, incorporating a healthy diet is critical to clinical care [1]. Acknowledging the slight differences between the four effective dietary approaches is essential. For instance, many of the interventions included recommendations to consume fiber-rich foods, whole grain products, and limit sugar-sweetened beverages. Consequently, it would be advisable for physicians to guide patients towards adopting a healthy diet that aligns with their personal preferences. By focusing on sustainable dietary modifications that are compatible with personal choices, patients are more likely to achieve long-term adherence and experience the benefits of a healthier diet. It is important to provide guidance and support to help patients make informed decisions and establish dietary habits that promote their overall well-being.

### Strength and Limitation

Although our study is not the first network meta-analysis to assess the comparative effects of different dietary approaches on glycemic control in T2DM patients, we made some further exploration. First, our research is the network meta-analysis with the largest number of dietary patterns compared [28]. Second, to explore the long-term effects of dietary approaches on glycemic control, we set the minimum study duration to 6 months, while other studies which focused on a similar objective, setting their criteria at 12 weeks [25] or no study length limitation [26,111]. The duration of the study length was a key factor in dietary as well as lifestyle intervention studies, as the effect and adherence declined over time [25]. Third, recommended diets, in which a specific diet was recommended to patients in the control group (e.g., advice based on ADA guideline), was included as a comparison, which was commonly mixed with the usual diet/no intervention in the previous meta-analysis. Our refined division made our dietary patterns more comprehensive and complete than other reviews. Last but not least, we included the ketogenic diet, quite interesting to the public but controversial, as one of the included dietary patterns, and found the effects on glycemic control, which no published systematic reviews have explored yet. 

Some limitations of our study need to be acknowledged. First, regarding the quality of the included literature, the studies included in our network meta-analysis were mainly of very low to moderate quality, partly due to the lack of allocation concealment and blinding. However, this problem is indeed difficult to avoid in randomized controlled trials of dietary patterns, we adjusted our assessment for overall bias if the studies only risked blinding. Moreover, we did a sensitivity analysis to remove high risk of bias studies. The effectiveness of the ketogenic diet and moderate carbohydrate diet on HbA1c and fasting glucose remained, confirming the results we obtained are robust. Second, we found heterogeneity in the outcome of HbA1c. Therefore, we conducted univariate meta-regression analyses to investigate the association between differences in weight change and reductions in glycemic indexes, as the source of heterogeneity. However, not all of the included studies reported data on body weight change, which may generate bias toward the overall effect. Third, all of the included studies did not describe whether participants actually followed the dietary approach in the protocol design and did not collect data on the daily intake of each dietary component, thus potentially influencing the actual effect on outcome indicators. Forth, we must acknowledge that the number of long-term studies for glycemic control in T2DM patients is limited, and the findings need to be considered in the light of very low to moderate credibility of evidence. Large-scale, long-term, well-designed randomized trials are needed to assess further the long-term safety, efficacy and compliance of dietary approaches on T2DM patients. Our study primarily focused on glycemic control; however, we acknowledge the importance of examining adherence and compliance to the dietary approaches. These factors are highly relevant to patients’ decision-making, regarding their preferred dietary approach and the implementation in real-world clinical practice. Paying closer attention to adherence and compliance can provide valuable insights for improving patient outcomes and tailoring dietary recommendations to individual needs.

## 5. Conclusions

In summary, T2DM patients, following dietary approaches, including the ketogenic diet, Mediterranean diet, moderate-carbohydrate diet, and low glycemic index diet, experienced significant improvements in glycemia. Although this study found the ketogenic diet superior, further high-quality and long-term studies are needed to strengthen its credibility. 

## Figures and Tables

**Table 1:** Characteristic of included trials.

Study, Country	*n*	Duration (Months)	% of Female	RCT Condition ^a^			Outcome(s)	Energy Restriction	Exercise	Diabetes Medications	Drop-Out	Adverse Event(s)
				1	2	3						
Uusitupaa 1993 [54], Sweden	86	12	43	LF (50.7/53.7) ^b^	CON (54.0/54.4) ^b^	—	HbA1c, FG	Yes.	No.	Yes.	0%	/
Milne 1994 [55], New Zealand	64	18	54.7	MC (59)	LF (60)	CON (58)	HbA1c	Yes.	No.	Yes.	8.5%	/
Brinkworth 2004 [56], Australia	38	16	60.5	HP (60.9)	LF (62.7)	—	HbA1c, FG	No.	/	Yes.	40.6%	/
Westman 2008 [58], USA	50	6	79	KD (51.2)	LGI (50.0)	—	HbA1c, FG	Yes.	No.	Yes.	40.5%	Headache, constipation, diarrhea, insomnia, and back pain (*p* > 0.05).
Wolever 2008 [59], Canada	156	12	54	LF (60.4)	LGI (60.6)	MC (58.6)	HbA1c	Yes.	Yes.	Yes.	19.75%	2 adverse events in LF and MC, respectively.
Ma 2008 [57], USA	40	12	53	REC (53) ^c^	LGI	—	HbA1c	No.	No.	Yes.	0%	/
Barnard 2009 [60], USA	99	18.5	60.6	VEG (56.7)	REC (54.6)	—	HbA1c, FG	Yes.	No.	Yes.	28.3%	No adverse events.
Brehm 2008 [61], USA	95	12	62.9	MC (56.5) ^c^	LF	—	HbA1c, FG	Yes.	No.	/	23%	/
Esposito 2009 [63], Italy	215	48	50.6	MD (52.4)	LF (51.9)	—	HbA1c, FG	Yes.	Yes.	Yes.	9.3%	21% in MD and 23% in LF reported at least 1 adverse event.
Davis 2009 [62], USA	105	12	78.1	LC (54)	LF (53)	—	HbA1c	No.	No.	Yes.	13.33%	/
Neelima 2009 [64], USA	89	36	/	LF (/)	CON (/)	—	HbA1c	/	/	/	/	/
Elhayany 2010 [66], Israel	194	12	44.3	REC (55) ^c^	MD	MC	HbA1c, FG	No.	No.	/	30.9%	/
Iqbal 2010 [68], USA	68	24	10.4	LC (59.4) ^c^	LF	—	HbA1c, FG	Yes.	No.	Yes.	52.78%	No adverse events.
Coppell 2010 [65], New Zealand	93	6	59.1	LF (56.6)	CON (58.4)	—	HbA1c, FG	Yes.	Yes.	Yes.	9.62%	/
Huang 2010 [67], Taiwan, China	154	12	56.5	LF (56.6)	CON (56.9)	—	HbA1c, FG	No.	No.	Yes.	20.2%	/
Nystrom 2011 [73], Sweden	61	24	/	LF (/)	LC (/)	—	HbA1c	Yes.	/	/	0%	/
Goldstein 2011 [71], Israel	30	12	48.1	LC (57)	REC (55)	—	HbA1c, FG	LC: No. REC: Yes.	No.	/	42.3%	/
Kahleova 2011 [72], Czech Republic	74	6	52.7	VEG (54.6)	REC (57.7)	—	HbA1c, FG	Yes.	Yes.	Yes.	32.4%	/
Fabricatore 2011 [70], USA	79	10	79.7	LF (52.5)	LGI (52.8)	—	HbA1c, FG	Yes.	Yes.	/	36.7%	/
Andrews 2011 [69], UK	347	12	36.6	LF (60.1)	CON (59.5)	—	HbA1c	LF: Yes. CON: No.	No.	Yes.	2.3%	/
Guldbrand 2012 [74], Sweden	61	24	55.8	LC (62.7)	LF (61.2)	—	HbA1c	Yes.	/	Yes.	0%	/
Krebs 2012 [75], New Zealand	419	24	60	HP (57.7)	LF (58.0)	—	HbA1c, FG	Yes.	/	Yes.	30%	/
Timar 2013 [76], Romania	223	12	/	MD (/)	REC (/)	CON (/)	HbA1c	MD: Yes. REC: Yes. CON: No.	No.	Yes.	/	/
Pedersen 2014 [78], Australia	64	12	22.2	HP (59.4)	LF (62.4)	—	HbA1c, FG	Yes.	/	Yes.	29.7%	No adverse events.
Tay 2014 [79], USA	78	13	42.6	LC (/)	LF (/)	—	HbA1c, FG	Yes.	Yes.	/	32%	/
Yamada 2014 [80], Japan	24	6	50	LC (63.3)	LF (63.2)	—	HbA1c, FG	LC: No. LF: Yes.	/	/	0%	/
Lasa 2014 [77], Spain	141	12	59.7	MD (67.4)	LF (67.2)	—	FG	No.	No.	/	0%	No adverse effects.
Rock 2014 [44], USA	227	12	51.1	LF (55.5)	MC (57.3)	CON (56.8)	HbA1c, FG	Yes.	No.	Yes.	10%	/
Bahado-Singh 2015 [81], Jamaica	65	6	55	LGI (42.5)	CON (43.0)	—	HbA1c, FG	/	/	/	18.5%	/
Liu 2015 [82], China	117	12	60.7	LF (63.3)	CON (62.0)	—	HbA1c, FG	/	No.	/	0%	/
Watson 2016 [83], Australia	61	6	45.9	HP (54)	LF (55)	—	HbA1c, FG	Yes.	Yes.	Yes.	27.9%	/
Wycherley 2016 [84], Australia	115	12	42.6	LC (58.4) ^c^	LF	—	HbA1c	Yes.	Yes.	/	32.1%	/
Sato 2016 [86], Japan	62	6	24.2	LC (60.5)	REC (58.4)	—	HbA1c	No.	No.	Yes.	6.1%	/
Maiorino 2016 [85], Italy	201	42	50.7	MD (52.4)	LF (51.9)	—	HbA1c, FG	Yes.	No.	/	9.3%	/
Pavithran 2019 [87], India	30	6	46.7	LGI (52) ^c^	CON	—	HbA1c	/	/	/	/	/
Pavithran 2020 [91], India	36	6	41.7	LGI (52) ^c^	CON	—	HbA1c	/	/	/	10%	/
Chen 2020 [88], Taiwan, China	85	18	61.1	LC (63.1)	REC (64.1)	—	HbA1c, FG	No.	No.	Yes.	7.6%	No adverse effects on lipid profiles.
Gutierrez-Mariscal 2020 [89], Spain	183	60	16.9	MD (60.3)	LF (59.9)	—	HbA1c, FG	No.	No.	/	2.2%	/
Marco-Benedi 2020 [90], Spain	73	6	56.2	HP (56.6)	LF (54.5)	—	HbA1c, FG	Yes.	Yes.	Yes.	8.2%	/
Kakoschke 2021 [92], Australia	115	48	42.6	LC (58.5) ^c^	LF	—	HbA1c	Yes.	Yes.	/	47%	/
Zahedi 2021 [93], Iran	228	6	77.2	MD (57.3) ^c^	CON	—	HbA1c, FG	/	/	/	7.9%	/
Gram-Kampmann 2022 [94], Denmark	64	6	56.3	LC (57.3)	REC (55.2)	—	HbA1c, FG	No.	No.	Yes.	9.8%	An increased frequency of gastrointestinal complaints (*p* = 0.03) such as constipation (*n* = 5), diarrhea (*n* = 2), and abdominal discomfort (*n* = 3) was found in LC group

Note. ^a^ Values in parentheses represent mean ages of participants in each RCT condition. ^b^ Ages reported as men/women. ^c^ Age for all participants. HbA1c = Hemoglobin A1c, FG = Fasting glucose. RCT conditions: LC = low-carbohydrate, MC = moderate-carbohydrate, KD = ketogenic, LF = low-fat, HP = high-Protein, MD = Mediterranean, VEG = vegetarian/vegan, LGI = low GI/GL, REC = recommended, and CON = control. Recommendation included advice(s) based on American Diabetes Association (ADA), conventional/traditional diabetic diet(s), standard diabetes diet(s), recommended nutrition therapy(s) or the Danish dietary guideline. “—” means not applicable while “/” means not reported.

**Table 2:** League table comparing the effects of all dietary approaches for HbA1c (%) and fasting glucose (mmol/L), respectively.

Fasting Glucose (mmol/L)									
**KD**	−0.53 (−2.86,1.79)	−0.21 (−2.28,1.85)	−0.86 (−3.46,1.75)	−0.18 (−2.53,2.17)	−1.23 (−3.63,1.18)	−0.59 (−2.95,1.77)	−0.78 (−3.18,1.62)	−0.72 (−3.03,1.58)	−1.48 (−3.77,0.82)
−0.86 (−2.06,0.34)	**MD**	0.32 (−0.75,1.39)	−0.32 (−1.57,0.93)	0.35 (−0.25,0.95)	−0.69 (−1.44,0.06)	−0.06 (−0.71,0.59)	−0.25 (−1.01,0.51)	−0.19 (−0.58,0.20)	**−0.95 (−1.51,−0.38)**
−1.00 (−2.05,0.05)	−0.14 (−0.73,0.45)	**LGI**	−0.64 (−2.23,0.95)	0.03 (−1.08,1.15)	−1.01 (−2.25,0.22)	−0.37 (−1.51,0.76)	−0.57 (−1.79,0.66)	−0.51 (−1.52,0.50)	**−1.26 (−2.26,−0.27)**
−1.01 (−2.21,0.20)	−0.15 (−0.80,0.50)	−0.01 (−0.60,0.59)	**VEG**	0.68 (−0.58,1.94)	−0.37 (−1.37,0.63)	0.27 (−1.08,1.62)	0.08 (−1.12,1.27)	0.13 (−1.11,1.38)	−0.62 (−1.93,0.68)
−1.09 (−2.29,0.11)	−0.23 (−0.87,0.42)	−0.09 (−0.68,0.50)	−0.08 (−0.73,0.57)	**MC**	**−1.04 (−1.81,−0.28)**	−0.41 (−1.18,0.36)	−0.60 (−1.38,0.18)	−0.54 (−1.11,0.02)	**−1.30 (−1.92,−0.67)**
−1.33 (−2.48,−0.19)	−0.47 (−1.00,0.06)	−0.33 (−0.79,0.13)	−0.32 (−0.71,0.06)	−0.24 (−0.77,0.28)	**RECOM**	0.64 (−0.27,1.54)	0.44 (−0.20,1.09)	0.50 (−0.24,1.24)	−0.25 (−1.09,0.58)
**−1.40 (−2.62,−0.17)**	−0.53 (−1.22,0.15)	−0.40 (−1.03,0.24)	−0.39 (−1.08,0.31)	−0.31 (−0.82,0.20)	−0.06 (−0.64,0.52)	**HP**	−0.19 (−1.07,0.69)	−0.13 (−0.65,0.38)	**−0.89 (−1.60,−0.18)**
**−1.49 (−2.71,−0.27)**	−0.63 (−1.30,0.05)	−0.49 (−1.11,0.13)	−0.48 (−1.16,0.20)	−0.40 (−0.90,0.09)	−0.16 (−0.72,0.41)	−0.09 (−0.39,0.20)	**LC**	0.06 (−0.65,0.77)	−0.70 (−1.52,0.13)
**−1.45 (−2.66,−0.25)**	−0.59 (−1.24,0.06)	−0.45 (−1.05,0.14)	−0.45 (−1.10,0.21)	−0.37 (−0.82,0.09)	−0.12 (−0.66,0.41)	−0.06 (−0.28,0.17)	0.04 (−0.15,0.22)	**LF**	**−0.75 (−1.24,−0.27)**
**−0.73 (−1.19,−0.28)**	−0.47 (−1.27,0.34)	−0.37 (−0.83,0.10)	−0.48 (−3.10,2.15)	−0.33 (−0.83,0.17)	−0.43 (−0.94,0.09)	0.06 (−0.45,0.57)	**−0.69 (−1.32,−0.06)**	**−1.82 (−2.93,−0.71)**	**CON**
HbA1c (%)									

Note. Labels on the diagonal represent the RCT conditions, KD = ketogenic, MD = Mediterranean, LGI = low GI/GL, VEG = vegetarian/vegan, MC = moderate-carbohydrate, RECOM = recommended, HP = high-protein, LC = low-carbohydrate, LF = low-fat, CON = Control. Off-diagonal values represent mean differences in the reduction of HbA1c (below diagonal) and fasting glucose (above diagonal) for any pair of combination of the RCT conditions, followed by the corresponding 95% confidence intervals (in parentheses). For illustration, the mean difference in average HbA1c between the ketogenic and control diet is −0.73%. Statistically significant treatment effects are in bold.

**Table 3:** SUCRA ranking for the dietary approaches.

	HbA1c	SUCRA (%)	Fasting Glucose	SUCRA (%)
1	Ketogenic	97.5	Moderate-carbohydrate	82.7
2	Mediterranean	78.1	Low GI/GL	75.4
3	Low GI/GL	69.0	Ketogenic	71.0
4	Vegetarian/Vegan	68.9	Mediterranean	61.3
5	Moderate-carbohydrate	62.7	High-protein	56.3
6	Recommended	38.9	Low-fat	44.2
7	High-protein	35.5	Low-carbohydrate	44.1
8	Low-carbohydrate	25.3	Vegetarian/Vegan	41.6
9	Low-fat	22.5	Recommended	16.3
10	Control	1.7	Control	6.9

## Data Availability

Data are available upon request.

## Supplementary Materials

The following supporting information can be downloaded at: https://www.mdpi.com/article/10.3390/nu15143156/s1, File S1: Searching Strategy; Figures S1 and S2: Interval plots; Figures S3 and S4: SUCRA; Figure S5: Meta-regression for HbA1c; Figure S6: Meta-regression for fasting glucose; Figures S7 and S8: Comparison-adjusted funnel plot; Figure S9 and S10: Bar graphs; Table S1: Full text articles excluded; Table S2: Specific study characteristics; Table S3: Details for included recommended diets; Tables S4 and S5: Side-splitting approach; Tables S6–S19: Subgroup and sensitivity analysis; Tables S20 and S21: GRADE assessment; Table S22: Mean differences in body weight at 6 and 12 months intervention; Table S23: SUCRA ranking for the dietary approaches on weight change. References [55,72,86,112,113,114,115,116,117,118,119,120,121,122,123,124,125,126,127,128,129,130,131,132,133,134,135,136,137,138,139,140,141,142,143,144,145,146,147,148,149,150,151,152,153,154,155,156,157,158,159,160,161,162,163] are cited in the supplementary materials.
