# Supplementary material for: Effect of Dietary Approaches on Glycemic Control in Patients with Type 2 Diabetes: A Systematic Review with Network Meta-Analysis of Randomized Trials
Source: Nutrients. 2023 Jul 15;15(14):3156. doi: 10.3390/nu15143156 (PMC10384204; doi:10.3390/nu15143156)
Supplement: Supplementary file 1 [file nutrients-15-03156-s001.zip › nutrients-2476483-supplementary.pdf]

Supplementary File S1: Searching Strategy
Supplementary Table S1: Full text articles excluded
Supplementary Table S2: Specific study characteristics
Supplementary Table S3: Details for included recommended diets
Supplementary Figures S1-S2: Interval plots
Supplementary Tables S4-S5: Side-splitting approach
Supplementary Figures S3-S4: SUCRA
Supplementary Tables S6-S19: Subgroup and sensitivity analysis
Supplementary Figure S5: Meta-regression for HbA1c
Supplementary Figure S6: Meta-regression for fasting glucose
Supplementary Figures S7-S8: Comparison-adjusted funnel plot
Supplementary Figures S9-S10: Bar graphs
Supplementary Tables S20-S21: GRADE assessment
Supplementary Table S22: Mean differences in body weight at 6 and 12 months intervention
Supplementary Table S23: SUCRA ranking for the dietary approaches on weight change
Supplementary References of excluded studies

Supplementary File S1: Full search strategy

Pubmed/Medline

#1 (randomized controlled trial [pt] OR controlled clinical trial [pt] OR randomized [tiab] OR placebo [tiab] OR clinical trials as topic [mesh: noexp] OR randomly [tiab] OR trial [ti]) NOT (animals [mh] NOT humans [mh])

#2 "Diabetes Mellitus"[Mesh]

#3 (((((((diabet*[Title/Abstract]) OR (IDDM[Title/Abstract])) OR (NIDDM[Title/Abstract])) OR (MODY[Title/Abstract])) OR (T1DM[Title/Abstract])) OR (T2DM[Title/Abstract])) OR (T1D[Title/Abstract])) OR (T2D[Title/Abstract])

#4 #2 OR #3

#5 "portfolio diet*"[Title/Abstract] OR "dietary portfolio"[Title/Abstract]

#6 low[Title/Abstract] AND (diet*[Title/Abstract] AND ("gi"[Title/Abstract] OR "glycemic index"[Title/Abstract] OR "glycemic load"[Title/Abstract] OR "glycemic indices"[Title/Abstract]))

#7 ("Ornish diet"[Title/Abstract] OR "vegetarianism"[Title/Abstract] OR "plant based diet"[Title/Abstract] OR "lacto vegetarian"[Title/Abstract] OR "lacto-ovo vegetarian"[Title/Abstract] OR "diet vegetarian"[Title/Abstract] OR "diet, vegetarian"[MeSH Terms] OR "diet, vegan"[MeSH Terms])

#8 ("dash"[Title/Abstract]) OR ("Dietary Approaches To Stop Hypertension"[Title/Abstract]) OR ("Dietary Approaches To Stop Hypertension"[Mesh])

#9 (nordic AND diet*) OR (nordic diet*) OR ("baltic sea" AND diet*)

#10 ("Diet, Paleolithic"[Mesh]) OR ((((((((((Paleolithic[Title/Abstract] AND diet*)) OR ((Paleo[Title/Abstract] AND diet*)) OR ((Paleo[Title/Abstract] AND nutrition*))) OR ((Paleolithic[Title/Abstract] AND nutrition))) OR (("stone age"[Title/Abstract] AND diet*))) OR (("stone age"[Title/Abstract] AND nutrition*))) OR ((caveman[Title/Abstract] AND diet*))) OR ((caveman[Title/Abstract] AND nutrition*))) OR (("Hunter-Gatherer"[Title/Abstract] AND diet*))) OR (("Hunter-Gatherer"[Title/Abstract] AND nutrition*))))

#11 ((((("Mediterranean diet"[Title/Abstract]) OR ("Mediterranean dietary pattern*"[Title/Abstract])) OR (MedDiet[Title/Abstract])) OR ("MeDiet"[Title/Abstract])) OR ("cretan diet"[Title/Abstract])) OR ("Diet, Mediterranean"[Mesh])

#12 ("high protein diet*"[Title/Abstract]) OR ("Diet, High-Protein"[Mesh])

#13 ("Diet, Fat-Restricted"[Mesh]) OR (('low fat'[Title/Abstract] OR 'fat free'[Title/Abstract] OR fat-restricted [Title/Abstract] OR "fat restriction"[Title/Abstract] OR "restricted fat"[Title/Abstract]) AND diet*[Title/Abstract])

#14 (((ketogenic diet[Title/Abstract]) OR (Ketosis[Title/Abstract])) OR ("Diet, Ketogenic"[Mesh]))

#15 "Diet, Carbohydrate-Restricted"[Mesh]

#16 (("low carb*"[Title/Abstract]) AND diet*[Title/Abstract]) OR "atkins diet"[Title/Abstract] OR "modified atkins diet"[Title/Abstract] OR "high-protein low-carbohydrate diet"[Title/Abstract] OR "very low carbohydrate diet"[Title/Abstract] OR "very low-carbohydrate diet"[Title/Abstract] OR "low CHO diet"[Title/Abstract] OR "carbohydrate restricted diet"[Title/Abstract] OR "CHO restricted diet"[Title/Abstract]

#17 #15 OR #16
#18 #5 OR #6 OR #7 OR #8 OR #9 OR #10 OR #11 OR #12 OR #13 OR #14 OR #17
#19 #18 AND #1 AND #4

Embase
#31 #11 AND #14 AND #30
#30 #15 OR #18 OR #21 OR #22 OR #23 OR #24 OR #25 OR #26 OR #27 OR #28 OR #29
#29 'low carbohydrate diet'/exp OR (('low carb*' NEAR/1 diet*):ab,ti) OR (('carb* restrict*' NEAR/1 diet*):ab,ti) OR 'atkins diet':ab,ti OR 'modified atkins diet':ab,ti OR 'high-protein low-carbohydrate diet':ab,ti OR 'very low carbohydrate diet':ab,ti OR 'very low-carbohydrate diet':ab,ti OR 'low cho diet*':ab,ti OR 'cho restricted diet*':ab,ti
#28 'ketogenic diet'/exp OR 'ketogenic diet*':ab,ti OR ((keto* NEAR/1 diet*):ab,ti) OR ketosis:ab,ti
#27 'low fat diet'/exp OR (('low fat' NEAR/3 diet*):ab,ti) OR (('fat free' NEAR/3 diet*):ab,ti) OR (('fat-restricted' NEAR/3 diet*):ab,ti) OR (('fat restriction' NEAR/3 diet*):ab,ti) OR (('restricted fat' NEAR/3 diet*):ab,ti)
#26 'protein diet'/exp OR 'high protein diet*':ab,ti
#25 'mediterranean diet'/exp OR 'mediterranean diet*':ab,ti OR (('mediterranean diet*' NEXT/2 pattern*):ab,ti) OR meddiet:ab,ti OR mediet:ab,ti OR 'cretan diet':ab,ti
#24 'paleolithic diet'/exp OR ((paleolithic NEXT/1 diet*):ab,ti) OR ((paleolithic NEXT/1 nutrition*):ab,ti) OR ((paleo NEXT/1 diet*):ab,ti) OR ((paleo NEXT/1 nutrition*):ab,ti) OR (('stone age' NEXT/1 diet*):ab,ti) OR (('stone age' NEXT/1 nutrition*):ab,ti) OR ((caveman NEXT/1 diet*):ab,ti) OR ((caveman NEXT/1 nutrition*):ab,ti) OR (('hunter-gatherer' NEXT/1 diet*):ab,ti) OR (('hunter-gatherer' NEXT/1 nutrition*):ab,ti) OR 'no-grain diet*':ab,ti OR 'grain-free diet*':ab,ti OR 'primal diet*':ab,ti OR 'evolution diet*':ab,ti OR 'primitive diet*':ab,ti OR 'ancestral human diet*':ab,ti
#23 'nordic diet'/exp OR (('nordic' NEXT/1 diet*):ab,ti) OR (('baltic sea' NEXT/1 diet*):ab,ti)
#22 'dash diet'/exp OR 'dash diet*':ab,ti OR 'dietary approach* to stop* hypertension':ab,ti
#21 #19 OR #20
#20 'vegetarian diet'/exp OR 'vegan diet'/exp OR 'plant based diet'/exp OR 'lacto vegetarian'/exp OR 'lacto-ovo vegetarian'/exp
#19 'chip diet':ab,ti OR 'ornish diet*':ab,ti OR 'vegetarianism':ab,ti OR 'plant based diet':ab,ti OR 'lacto vegetarian':ab,ti OR 'lacto-ovo vegetarian':ab,ti OR 'vegetarian diet*':ab,ti
#18 #16 OR #17
#17 low:ab,ti AND ((('gi' OR 'glycemic index' OR 'glycemic load' OR 'glycemic indices') NEAR/1 diet*):ab,ti)
#16 'low glycemic index diet'/exp
#15 'portfolio diet*':ab,ti OR 'diet* portfolio':ab,ti
#14 #12 OR #13

#13 diabet*:ab,ti OR iddm:ab,ti OR niddm:ab,ti OR mody:ab,ti OR t1dm:ab,ti OR t2dm:ab,ti OR t1d:ab,ti OR t2d:ab,ti
#12 'diabetes'/exp
#11 #1 OR #2 OR #3 OR #4 OR #5 OR #6 OR #7 OR #8 OR #9 OR #10
#10 (singl*:ab,ti OR doubl*:ab,ti OR trebl*:ab,ti OR tripl*:ab,ti) AND (blind*:ab,ti OR mask*:ab,ti)
#9 random*:ab,ti AND (allocat*:ab,ti OR assign*:ab,ti OR order*:ab,ti)
#8 (clinical:ab,ti OR control*:ab,ti OR comparativ*:ab,ti OR prospectiv*:ab,ti OR randomi?ed:ab,ti) AND (trial*:ab,ti OR stud*:ab,ti)
#7 'comparative study'/exp
#6 'clinical trial'/exp
#5 'controlled clinical trial'/exp
#4 'single blind procedure'/exp
#3 'double blind procedure'/exp
#2 'randomization'/exp
#1 'randomized controlled trial'/exp

Cochrane CENTRAL
#1 MeSH descriptor: [Diabetes Mellitus] explode all trees
#2 (diabet* OR iddm OR niddm OR mody OR t1dm OR t2dm OR t1d OR t2d):ti,ab,kw
#3 #1 OR #2
#4 (randomized controlled trial):pt
#5 (controlled clinical trial):pt
#6 (randomi?ed):ti,ab,kw
#7 (placebo):ti,ab,kw
#8 (randomly):ti,ab,kw
#9 (trial):ti,ab,kw
#10 (groups):ti,ab,kw
#11 #4 OR #5 OR #6 OR #7 OR #8 OR #9 OR #10
#12 ('diet* NEAR/1 portfolio'):ti,ab,kw
#13 (('gi' NEAR/1 diet*) OR ('glycemic index' NEAR/1 diet*) OR ('glycemic load' NEAR/1 diet*) OR ('glycemic indices' NEAR/1 diet*)):ti,ab,kw AND (low):ti,ab,kw
#14 MeSH descriptor: [Diet, Vegetarian] explode all trees
#15 MeSH descriptor: [Diet, Vegan] explode all trees
#16 ('chip diet' OR 'ornish diet*' OR 'vegetarianism' OR 'plant based diet' OR 'lacto vegetarian' OR 'lacto-ovo vegetarian' OR 'vegetarian diet*'):ti,ab,kw
#17 #14 OR #15 OR #16
#18 MeSH descriptor: [Dietary Approaches To Stop Hypertension] explode all trees
#19 ('dash diet*' OR 'dietary approach* to stop* hypertension'):ti,ab,kw
#20 #18 OR #19
#21 (('nordic' NEXT/1 diet*) OR ('baltic sea' NEXT/1 diet*)):ti,ab,kw
#22 MeSH descriptor: [Diet, Paleolithic] explode all trees

#23 ((paleolithic NEXT/1 diet*) OR (paleolithic NEXT/1 nutrition*) OR (paleo NEXT/1 diet*) OR (paleo NEXT/1 nutrition*) OR ('stone age' NEXT/1 diet*) OR ('stone age' NEXT/1 nutrition*) OR (caveman NEXT/1 diet*) OR (caveman NEXT/1 nutrition*) OR ('hunter-gatherer' NEXT/1 diet*) OR ('hunter-gatherer' NEXT/1 nutrition*) OR 'no-grain diet*' OR 'grain-free diet*' OR 'primal diet*' OR 'evolution diet*' OR 'primitive diet*' OR 'ancestral human diet*'):ti,ab,kw
#24 #22 OR #23
#25 MeSH descriptor: [Diet, Mediterranean] explode all trees
#26 ('mediterranean diet*' OR ('mediterranean diet*' NEXT/2 pattern*) OR meddiet OR mediet OR 'cretan diet'):ti,ab,kw
#27 #25 OR #26
#28 MeSH descriptor: [Diet, High-Protein] explode all trees
#29 ('high protein diet*'):ti,ab,kw
#30 #28 OR #29
#31 MeSH descriptor: [Diet, Fat-Restricted] explode all trees
#32 (('low fat' NEAR/3 diet*) OR ('fat free' NEAR/3 diet*) OR ('fat-restricted' NEAR/3 diet*) OR ('fat restriction' NEAR/3 diet*) OR ('restricted fat' NEAR/3 diet*)):ti,ab,kw
#33 #31 OR #32
#34 MeSH descriptor: [Diet, Ketogenic] explode all trees
#35 ('ketogenic diet*' OR (keto* NEAR/1 diet*) OR ketosis):ti,ab,kw
#36 #34 OR #35
#37 MeSH descriptor: [Diet, Carbohydrate-Restricted] explode all trees
#38 (('low carb*' NEAR/1 diet*) OR ('carb* restrict*' NEAR/1 diet*) OR 'atkins diet' OR 'modified atkins diet' OR 'high-protein low-carbohydrate diet' OR 'very low carbohydrate diet' OR 'very low-carbohydrate diet' OR 'low cho diet*' OR 'cho restricted diet*'):ti,ab,kw
#39 #37 OR #38
#40 #12 OR #13 OR #17 OR #20 OR #21 OR #24 OR #27 OR #30 OR #33 OR #36 OR #39
#41 #40 AND #3 AND #11

Web of knowledge
Title：
#1 (diabet* OR iddm OR niddm OR mody OR t1dm OR t2dm OR t1d OR t2d)
#2 (randomized OR randomly OR controlled OR groups OR trial)
#3 ('portfolio diet*' OR 'dietary portfolio')
#4 (('gi' OR 'glycemic index' OR 'glycemic index numbers' OR 'glycemic load' OR 'glycemic indices' OR 'glycemic index number') AND (diet*) AND (low))
#5 ('chip diet' OR 'ornish diet*' OR 'vegetarianism' OR 'plant based diet' OR 'lacto vegetarian' OR 'lacto-ovo vegetarian' OR 'vegetarian diet*')
#6 ('dash diet*' OR 'dietary approach* to stop* hypertension')
#7 (('nordic diet*') OR ('baltic sea diet*'))
#8 ('paleolithic diet*' OR 'paleolithic nutrition*' OR 'paleo diet*' OR 'paleo nutrition*' OR 'stone age diet*' OR 'stone age nutrition*' OR 'caveman diet*' OR 'caveman nutrition*' OR

'hunter-gatherer diet*' OR 'hunter-gatherer nutrition*' OR 'no-grain diet*' OR 'grain-free diet*' OR 'primal diet*' OR 'evolution diet*' OR 'primitive diet*' OR 'ancestral human diet*')
#9 ('mediterranean diet*' OR 'mediterranean diet* pattern*' OR meddiet OR mediet OR 'cretan diet')
#10 'high protein diet*'
#11 (('low fat' AND diet*) OR ('fat free' AND diet*) OR ('fat-restricted' AND diet*) OR ('fat restriction' AND diet*) OR ('restricted fat' AND diet*))
#12 ('ketogenic diet*' OR (keto* AND diet*) OR ketosis)
#13 (('low carb*' AND diet*) OR ('carb* restrict*' AND diet*) OR 'atkins diet' OR 'modified atkins diet' OR 'high-protein low-carbohydrate diet' OR 'very low carbohydrate diet' OR 'very low-carbohydrate diet' OR 'low cho diet*' OR 'cho restricted diet*')
#14 #13 OR #12 OR #11 OR #10 OR #9 OR #8 OR #7 OR #6 OR #5 OR #4 OR #3
#15 #14 AND #2 AND #1

ISRCTN
Text search:
diet* AND ('low carb*' OR 'low fat' OR 'high protein' OR mediterranean OR paleolithic OR nordic OR dash OR vegetarian OR gi OR portfolio) AND 'diabetes' AND (random OR trial OR groups)

Refine: adult, with results

ClinicalTrials.gov
Condition: Diabetes
Study type: interventional studies
Study results: with results
Age group: adult
Intervention: 'low carb*' OR 'low fat' OR 'high protein' OR mediterranean OR paleolithic OR nordic OR dash OR vegetarian OR gi OR portfolio

clinicaltrials register.eu
diet* AND ('low carb*' OR 'low fat' OR 'high protein' OR mediterranean OR paleolithic OR nordic OR dash OR vegetarian OR gi OR portfolio) AND 'diabetes' AND (random OR trial OR groups)
Advanced:
1、Select Age Range:adult
2、Results Status:trials with results

Supplemental Table S1. Characteristics of excluded studies (n = 55)

De Bont et al.,1981 [1]	No data available
Milne et al.,1994 [2]	Intervention not meeting inclusion criteria
Nielsen et al.,2005 [3]	Not a randomized trial
Fraser et al.,2008 [4]	No data available
Wolever et al.,2008 [5]	Duplicate
Haimoto et al.,2008 [6]	Not a randomized trial
Jenkins et al.,2008 [7]	Intervention not meeting inclusion criteria
Haimoto et al.,2009 [8]	Not a randomized trial
Moses et al.,2009 [9]	No data available
McGrievy et al.,2009 [10]	Duplicate
Kahleova et al.,2011 [11]	Duplicate
Cao et al.,2011 [12]	Intervention including medicine
Larsen et al.,2011 [13]	No data available
Shyam et al.,2011 [14]	No data available
Krebs et al.,2011 [15]	Duplicate
Tobias et al.,2012 [16]	Not a randomized trial
Davis et al.,2012 [17]	Intervention not meeting inclusion criteria
Jesudason et al.,2013 [18]	No data available
Shyam et al.,2013 [19]	Duplicate
Fernemark et al.,2013 [20]	Not a randomized trial
Shyam et al.,2013 [21]	Duplicate
Mayer et al.,2014 [22]	Intervention including medicine
Tay et al.,2014 [23]	Duplicate
Watson et al.,2015 [24]	Duplicate

Louie et al.,2015 [25]	Study length < 6 months
Zajac et al.,2016 [26]	Duplicate
Stentz et al.,2016 [27]	Duplicate
Wolever et al.,2017 [28]	Intervention not meeting inclusion criteria
Kahleova et al.,2017 [29]	Intervention not meeting inclusion criteria
Crowley et al.,2017 [30]	Intervention not meeting inclusion criteria
Benedi et al.,2017 [31]	Study length < 6 months
Krag et al.,2017 [32]	No data available
Sato et al.,2017 [33]	Duplicate
Zadeh et al.,2018 [34]	Intervention not meeting inclusion criteria
Tay et al.,2018 [35]	Duplicate
Torres et al.,2018 [36]	Population not meeting inclusion criteria
Björklund et al.,2018 [37]	No data available
Mattei et al.,2018 [38]	Population not meeting inclusion criteria
Crowder et al.,2019 [39]	Study length < 6 months
Mason et al.,2019 [40]	Intervention not meeting inclusion criteria
Stentz et al.,2019 [41]	No data available
Garbutt et al.,2020 [42]	Not a randomized trial
Kampmann et al.,2020 [43]	No data available
Tucker et al.,2020 [44]	No data available
Athinarayanan et al.,2020 [45]	No data available
Blindbak et al.,2020 [46]	No data available
Kobayashi et al.,2020 [47]	Intervention not meeting inclusion criteria
Tay et al.,2020 [48]	Duplicate
Ren et al.,2020 [49]	Study length < 6 months

Lawson et al.,2021 [50]	No data available
Buso et al.,2021 [51]	No data available
Dorans et al.,2021 [52]	No data available
Moriconi et al.,2021 [53]	Not a randomized trial
Han et al.,2021 [54]	No data available
Dimitra et al.,2022 [55]	No data available

Supplemental Table S2. Specific study characteristics of the included trials investigating the effects of different dietary approaches on Type 2 diabetes controlling

Study	Hypoglycaemic drugs (%)	Intervention under dietitian/nutritionists
Uusitupaa 1993	Except one man receiving glibenclamide in the CON group, all patients were treated with diet alone at the baseline.	No. Patient education was given by a doctor and two nurses trained in diabetes education.
Milne 1994	MC: Hypoglycemic drugs (50%)<br>LF: Hypoglycemic drugs (57%)<br>CON: Hypoglycemic drugs (52%)	Yes.
Brinkworth 2004	17 required oral hypoglycaemic medications;<br>3 required insulin	Yes.
Westman 2008	At baseline, 22 (75.9%) of the LGI group were taking hypoglycemic medications (insulin only *n* = 3, oral agents only n = 19), and 20 (95.2%) of the KD group were taking hypoglycemic medications (insulin + oral agents *n* = 4, insulin only *n* = 4, oral agents only *n* = 12)	Yes.
Wolever 2008	77 (48%) subjects were taking an angiotensin-converting enzyme inhibitor (*n* = 35), a diuretic (*n* = 26), a calcium channel blocker (*n* = 19), an angiotensin receptor blocker (*n* = 17), or β-blocker (*n* = 15) or an α-blocker (*n* = 5) (or both); 47 of the subjects were taking a single agent and 30 were taking ≥ 2 agents	Yes.
Ma 2008	Ninety percent (*n* = 36) were taking medication for diabetes. The most common oral medication was met formin (73%), followed by glyburide (38%). Ten subjects (25%) used insulin in addition to oral hypoglycemic agents.	Yes.
Barnard 2009	VEG: 69% on metformin; 51% on sulfonylurea; 33% on thiazolidinedione; 2% on other diabetes medications<br>REC: 78% on metformin; 58% on sulfonylurea; 30% on thiazolidinedione; 4% on other diabetes medications	Yes.
Brehm 2008	/	Yes.
Esposito 2009	Never been treated with antihyperglycemic drugs from the clinical practices of trial investigators.	Yes.
Davis 2009	LC: Metformin 78%, Sulfonylurea 44%, Insulin 35%<br>LF: Metformin 86%, Sulfonylurea 52%, Insulin 24%	Yes.
Neelima 2009	/	/

Elhayany 2010	/	Yes.
Iqbal 2010	Oral medications for diabetes:<br>LC: 84.3%, Sulfonylurea 57.1%, Metformin 61.4%, Thiazolidinedione 8.6%<br>LF: 85.1%, Sulfonylurea 43.2%, Metformin 52.7%, Thiazolidinedione 10.8%<br>Insulin for diabetes:<br>LC: 22.9%<br>LF: 29.7%	Yes.
Coppell 2010	LF: Oral hypoglycaemic agents only 69%, insulin only 2%, Insulin and oral hypoglycaemic agent(s) 29%<br>CON: Oral hypoglycaemic agents only 71%, insulin only 0%, Insulin and oral hypoglycaemic agent(s) 29%	Yes.
Huang 2010	LF: Sulfonylurea 54(96.4%), Bigrunide 45(80.4%), Thiazolidinedione 7(12.5%), Other oral hypoglycemia agent 0(0.0%)<br>CON: Sulfonylurea 57(95.0%), Bigrunide 50(83.3%), Thiazolidinedione 14(23.3%), Other oral hypoglycemia agent 2 (3.3%)	Yes.
Nystrom 2011	/	Yes.
Goldstein 2011	/	Yes.
Kahleova 2011	VEG: Metformin 29(78%), Sulphonylurea 20(54%), Thiazolidinedione 7(19%), Other 8(22%)<br>REC: Metformin 28(76%), Sulphonylurea 13(35%), Thiazolidinedione 5(14%), Other 3(8%)	Yes.
Fabricatore 2011	/	No. Doctoral- or masters-level clinicians training in clinical psychology.
Andrews 2011	CON: Metformin 32(32%), Sulphonylurea 7(7%), Thiazolidinedione 2(2%)<br>LF: Metformin 91(37%), Sulphonylurea 20(8%), Thiazolidinedione 1(0.4%)	Yes.
Guldbrand 2012	LF: 13(41.9%) using oral glucose-lowering medication only; 11(35.4%) using a combination of insulin and oral medication;<br>LC: 15(50%) using oral glucose-lowering medication only; 10(33.3%) using a combination of insulin and oral medication; 13 in the LF group and 15 in the LC	Yes.

	group were using oral glucose-lowering medication only, and 11 in the LF group and 10 in the LC group were treated with a combination of insulin and oral medication.	
Krebs 2012	HP: Oral agents only:56.0%<br>Insulin ± oral agents:24.6%<br>LF: Oral agents only:57.4%<br>Insulin ± oral agents:28.7%	Yes.
Timar 2013	Under Metformin, stabile dose monotherapy (100%)	/
Pedersen 2014	All other volunteers were treated with oral Blood glucose lowering medication and/or insulin (72/76) 94.74%<br>HP: 91%<br>LF: 92%<br>Of the 45 subjects who completed the study, 4 managed their diabetes with diet alone. Metformin in mono therapy was given to 17 volunteers, 10 were treated with a combination of metformin and sulfonylurea, 2 with metformin and glitazones, 3 with metformin, sulfonylurea and glitazones, 6 were treated with a combination of metformin and insulin glargine and 3 volunteers were treated with metformin, sulfonylurea and insulin Novomix and mixtard.	/
Tay 2014	/	/
Yamada 2014	/	Yes.
Lasa 2014	/	/
Rock 2014	LF: Insulin 19(25.7%); oral hypoglycemic 62(83.7%)<br>MC: Insulin 10(13.0%); oral hypoglycemic 69(89.6%)<br>CON: Insulin 12(15.8%); oral hypoglycemic 62(81.6%)	Yes.
Bahado-Singh 2015	/	/
Liu 2015	/	Yes.
Watson 2016	HP: Metformin 18(58%); Sulphonylureas 5(16%); GLP-1 Agonists 2(7%); DPP-4 Inhibitors 2(7%); Combination 3(10%); Insulin 6(19%)<br>LF: Metformin 18(64%); Sulphonylureas 5(18%); GLP-1 Agonists 2(7%); DPP-4 Inhibitors 3(11%); Combination 1(4%); Insulin 6(21%)	Yes.
Wycherley 2016	/	/
Sato 2016	REC: Basal supported oral therapy 1(3.1%); Intensive insulin therapy 13(40.6%); SU/sulfonylureas 14(44%);	Yes.

	Met/metformin 21(65.6%); TZD/thiazolidine 5(15.6%); DPP4i/Dipeptidyl peptidase-4 inhibitor 18(56.3%); a-GI/a-Glucosidase inhibitor 3(9.4%); GLP-1 receptor agonist 1(3.1%)<br>LC: Basal supported oral therapy 4(13.3%); Intensive insulin therapy 7(23.3%); SU/sulfonylureas 11(36.7%); Met/metformin 19(63.3%); TZD/thiazolidine 4(13.3%); DPP4i/Dipeptidyl peptidase-4 inhibitor 16(53.3%); a-GI/a-Glucosidase inhibitor 7(23.3%); Glinide 3(10%); GLP-1 receptor agonist 2(6.7%)	
Maiorino 2016	Specific medications not mentioned.	No. Nurses.
Pavithran 2019	/	/
Pavithran 2020	/	Yes.
Chen 2020	LC: oral hypoglycaemic agents 37(86.0%), Insulin or (OHA with insulin) 4(9.3%)<br>REC: oral hypoglycaemic agents 31(73.8%), Insulin or (OHA with insulin) 10(23.8%)	Yes.
Gutierrez-Mariscal 2020	/	/
Marco-Benedi 2020	LF: Metformin 12 (36.4%)<br>HP: Metformin 13 (35.1%)	Yes.
Kakoschke 2021	/	Yes.
Zahedi 2021	/	Yes.
Gram-Kampmann 2022	LC: Metformin 40(81.6%); Sulphonylurea 4(8.2%); DDP-4 inhibitor 6(12.2%); GLP-1 receptor analogue 9(18.4%); SGLT-2 inhibitor 10(20.4%); Insulin 2(4.1%)<br>REC: Metformin 16(72.7%); Sulphonylurea 0(0.0%); DDP-4 inhibitor 3(13.6%); GLP-1 receptor analogue 6(27.3%); SGLT-2 inhibitor 1(4.5%); Insulin 3(13.6%)	Yes.

Note. LC = low-carbohydrate, MC = moderate-carbohydrate, KD = ketogenic, LF = low-fat, HP = high-Protein, MED = Mediterranean, VEG = vegetarian/vegan, LGI = low GI/GL, REC = recommended, and CON = control.

Supplemental Table S3. Details for included recommended diets

Year	Author	Country	Recommended diet
2008	Ma	USA	Based on ADA recommendations; carbohydrates should provide 45% to 65% of total energy intake.
2009	Barnard	USA	Based on ADA recommendations; 15–20% protein, <7% saturated fat, 60–70% carbohydrate and monounsaturated fats; cholesterol ≤200 mg/d) was individualized, based on body weight and plasma lipid concentrations.
2010	Elhayany	Israel	Based on ADA recommendations; 50–55% carbohydrates, 30% fats and 20% proteins.
2011	Tori	Israel	Based on ADA recommendations; 10%–20% protein and the other 80% divided between fats [which provided 18–20% of calories as MUFA, 8–10% as polyunsaturated fatty acids (PUFA) and 9–10% as SFA], carbohydrates and 35 g of fiber. Men were allowed up to 1500 kcal/day and women, 1200 kcal/day.
2011	Kahleova	Czech	Based on DNSG recommendations; 50% carbohydrates, 20% protein, less than 30% fat (≤7% saturated fat, <200mg/day of cholesterol/day).
2013	Timar	Romania	Based on The Standard Diabetes Diet recommendations; 55% carbohydrates, 20% up to 30% fat and cholesterol (less than 7% from the total calories from saturated fat), 0.8 mg of proteins per kg daily and approximately 14 g of fiber per 1000 kcal. The cholesterol was limited to a maximum of 200 mg per day.
2016	Junko	Japan	Based on TDD recommendations; 50–60% carbohydrates, 1.0–1.2 g/kg of protein, the balance of the calories was covered by fat.
2020	Chin-Ying	Taiwan, China	Based on TDD recommendations; 50–60% carbohydrates, 1.0–1.2 g/kg of protein, and ≤ 30% for fat.
2022	Eva	Denmark	Based on the official Danish dietary guidelines; 50-60% carbohydrates mainly from fruit, vegetables, and whole-grain sources, 20-30% fat, where less than 10% should be from SFA, and 20-25% protein

Note. ADA = American Diabetes Association, DNSG = Diabetes and Nutrition Study Group, TDD = Traditional Diabetes Diet.

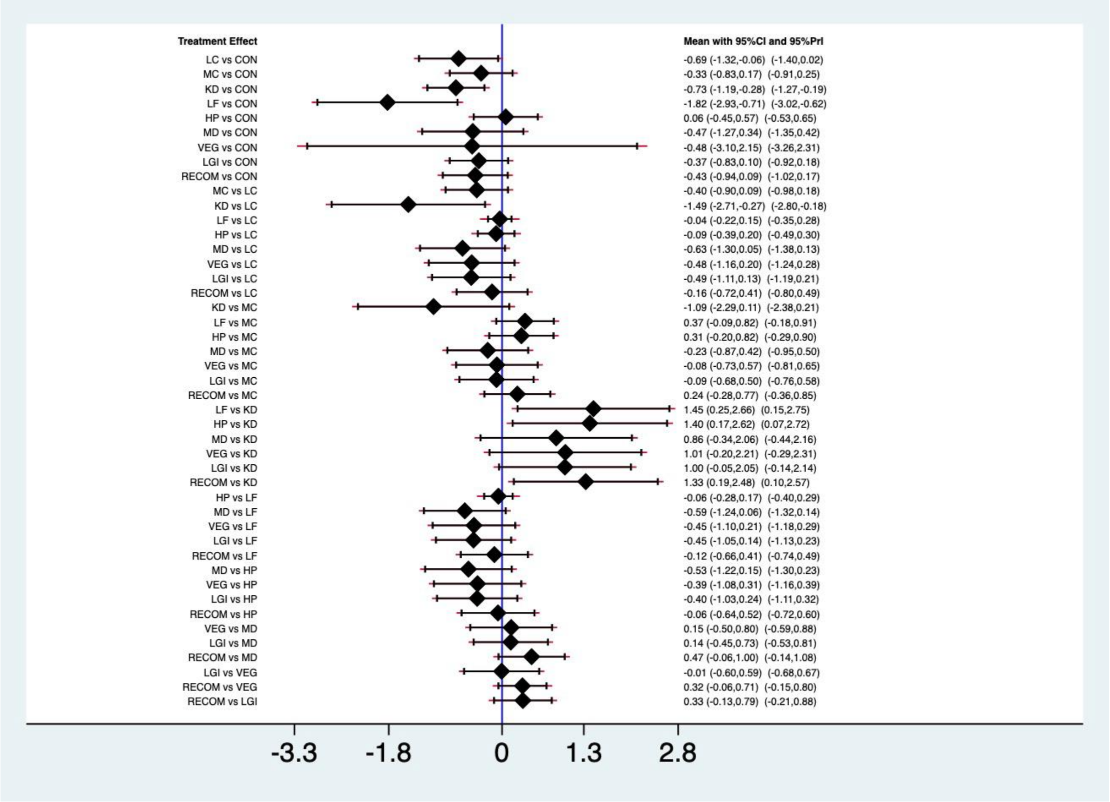

Supplemental Figure S1. Interval plot/ Contribution plot for this network meta-analysis.

Note. Mean differences for glycosylated haemoglobin (%) as estimated from network meta-analysis for every possible pair of interventions. Solid lines represent 95% CIs, and red lines 95% PrI. LC = low-carbohydrate, MC = moderate-carbohydrate, KD = ketogenic, LF = low-fat, HP = high-protein, MD = Mediterranean, VEG = Vegetarian/Vegan, LGI = low GI/GL, RECOM = recommended, CON = control. Predictive interval plot for HbA1c (%) network. The black lines represent the confidence intervals for the summary mean difference for each comparison and the red lines represent the respective predictive intervals (possible values in a future trial). The blue vertical line is the line of no effect (mean difference equal to 0).

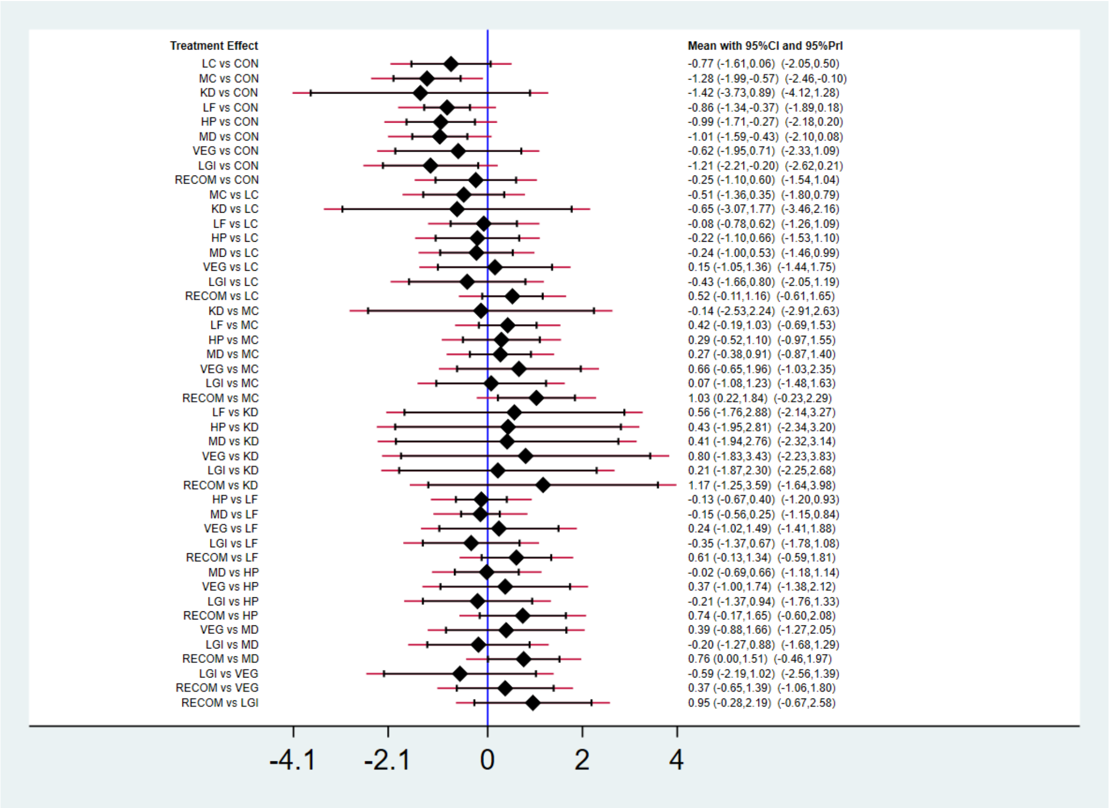

Supplemental Figure S2. Interval plot/ Contribution plot for this network meta-analysis

Note. Mean differences for fasting glucose (mmol/L) as estimated from network meta-analysis for every possible pair of interventions. Solid lines represent 95% CIs, and red lines 95% PrI. LC = low-carbohydrate, MC = moderate-carbohydrate, KD = ketogenic, LF = low-fat, HP = high-protein, MD = Mediterranean, VEG = Vegetarian/Vegan, LGI = low GI/GL, RECOM = recommended, CON = control. Predictive interval plot for fasting glucose (mmol/L) network. The black lines represent the confidence intervals for the summary mean difference for each comparison and the red lines represent the respective predictive intervals (possible values in a future trial). The blue vertical line is the line of no effect (mean difference equal to 0).

Side	Direct		Indirect		Difference		
	Coef.	Std. Err.	Coef.	Std. Err.	Coef.	Std. Err.	P>z
A C	-0.7039622	0.2845861	-0.6348009	0.2259702	-0.0691613	0.3632278	0.849
A E	-0.3237772	0.1223473	-0.5576592	0.1749064	0.233882	0.2135463	0.273
A G	-0.6226054	0.1855112	-0.5676062	0.1683261	-0.0549992	0.2513517	0.827
A I	-0.8369718	0.2199383	-0.7648704	0.2913257	-0.0721014	0.3652968	0.844
A J	-0.4911946	0.2216624	-0.2299169	0.1791081	-0.2612777	0.285076	0.359
B E	-0.0319665	0.0959179	0.7305537	0.1841504	-0.7625203	0.2136229	0.000
B J	0.5237335	0.1323448	-0.2387893	0.1571341	0.7625229	0.2136225	0.000
C E	0.1805792	0.2263032	0.3618025	0.2533706	-0.1812233	0.3404036	0.594
C G	0.1979237	0.2945958	-0.0046119	0.2242605	0.2025357	0.3669525	0.581
C I	-0.0857467	1.188055	-0.1510501	0.2397092	0.0653034	1.21215	0.957
C J	0.3895741	0.2953162	0.2869858	0.2453078	0.1025883	0.3814588	0.788
D I *	1	0.5640493	-1.461678	1185.328	2.461678	1185.328	0.998
E F *	-0.0631902	0.1462118	0.8088624	322.3802	-0.8720526	322.3803	0.998
E G	-0.2675618	0.1350663	-0.0762391	0.1681151	-0.1913227	0.2167379	0.377
E I	-0.6502896	0.342084	-0.3122464	0.217945	-0.3380433	0.4061844	0.405
G J	0.019097	0.1331875	0.7075936	0.1889214	-0.6884966	0.2364056	0.004
H J *	0.3232146	0.2327532	-0.6565505	508.6247	0.9797651	508.6248	0.998
I J	-0.08	0.4596154	0.614059	0.2259155	-0.694059	0.5121369	0.175

Supplemental Table S4. Evaluation of inconsistency for HbA1c (%) by using side-splitting approach heterogeneity estimates

Note. A = control diet, B = low-carbohydrate diet, C = moderate-carbohydrate diet, D = ketogenic diet, E = low-fat diet, F = high-protein diet, G = Mediterranean diet, H = Vegetarian/Vegan diet, I = low GI/GL diet, J = recommended diet.

Side	Direct		Indirect		Difference		
	Coef.	Std. Err.	Coef.	Std. Err.	Coef.	Std. Err.	P>z
A C	-1.239562	0.4453737	-1.358312	0.4684462	0.1187494	0.6473562	0.854
A E	-0.7472956	0.300332	-0.7794263	0.4592199	0.0321307	0.547856	0.953
A G	-1.0228	0.6518346	-0.923386	0.3294355	-0.099414	0.7303534	0.892
A I	-1.37	0.6614071	-1.106031	0.808146	-0.2639686	1.044299	0.800
B C	-0.1389026	0.8595359	-0.7211058	0.4448741	0.5822032	0.9678405	0.547
B E	-0.2748333	0.4970468	0.1960082	0.5320535	-0.4708415	0.7305975	0.519
B J	0.4850246	0.3912259	0.3495573	0.6431534	0.1354672	0.7522805	0.857
C E	0.5081262	0.4404325	0.563145	0.3927841	-0.0550188	0.5913667	0.926
C G	0.7909836	0.4811397	0.0700935	0.3867856	0.7208901	0.6172669	0.243
C J	1.226339	0.5149684	0.7864177	0.6073548	0.4399211	0.7946308	0.580
D I *	0.215	1.054263	-2.742407	632.6022	2.957407	632.6046	0.996
E F *	-0.1341753	0.2648504	1.368132	278.7903	-1.502308	278.7903	0.996
E G	-0.2788013	0.2198218	0.151029	0.4351681	-0.4298303	0.489054	0.379
E I	-0.3660019	0.7657319	-0.6299503	0.7100817	0.2639485	1.044299	0.800
G J	0.4196483	0.4911398	1.077494	0.5878636	-0.6578462	0.7629159	0.389
H J *	0.3693444	0.5120061	-0.8759375	447.0449	1.245282	447.0456	0.998

Supplemental Table S5. Evaluation of inconsistency for fasting glucose (mmol/L) by using side-splitting approach heterogeneity estimates

Note. A = control diet, B = low-carbohydrate diet, C = moderate-carbohydrate diet, D = ketogenic diet, E = low-fat diet, F = high-protein diet, G = Mediterranean diet, H = Vegetarian/Vegan diet, I = low GI/GL diet, J = recommended diet.

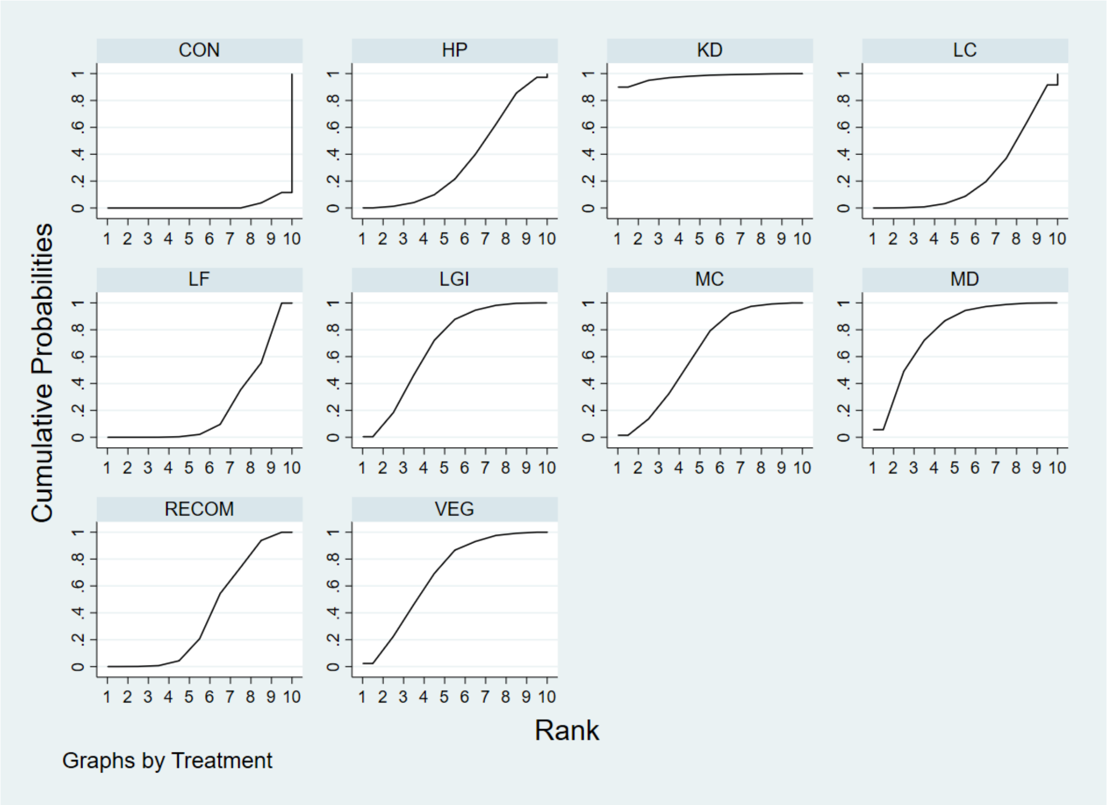

Supplemental Figure S3. SUCRA for HbA1c (%)

Note. LC = low-carbohydrate, MC = moderate-carbohydrate, KD = ketogenic, LF = low-fat, HP = high-protein, MD = Mediterranean, VEG = Vegetarian/Vegan, LGI = low GI/GL, RECOM = recommended, CON = control.

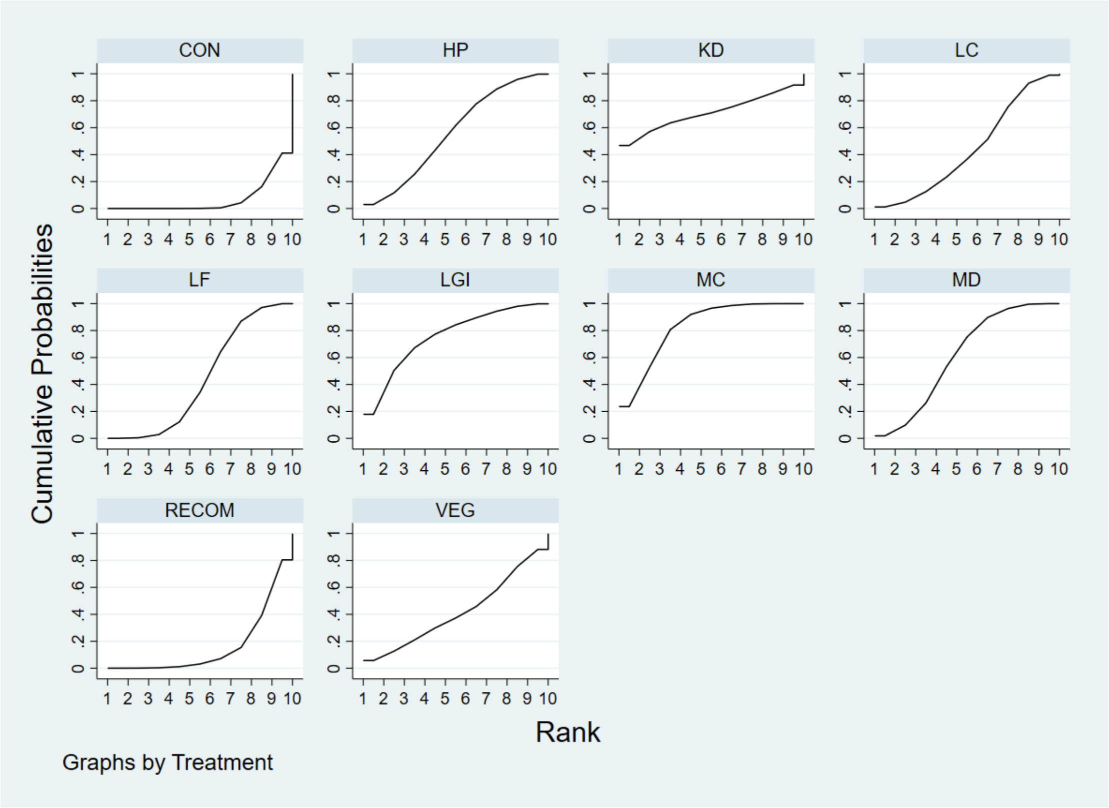

Supplemental Figure S4. SUCRA for fasting glucose (mmol/L)

Note. LC = low-carbohydrate, MC = moderate-carbohydrate, KD = ketogenic, LF = low-fat, HP = high-protein, MD = Mediterranean, VEG = Vegetarian/Vegan, LGI = low GI/GL, RECOM = recommended, CON = control.

**MC**								
-0.12 (-0.42,0.19)	**MD**							
-0.06 (-0.73,0.62)	0.06 (-0.57,0.69)	**VEG**						
-0.26 (-0.59,0.07)	-0.14 (-0.38,0.10)	-0.20 (-0.79,0.39)	**RECOM**					
-0.30 (-1.14,0.54)	-0.18 (-1.00,0.63)	-0.24 (-1.22,0.74)	-0.04 (-0.83,0.75)	**LGI**				
-0.28 (-0.72,0.15)	-0.16 (-0.53,0.20)	-0.22 (-0.94,0.49)	-0.02 (-0.43,0.38)	0.02 (-0.86,0.89)	**HP**			
-0.31 (-0.67,0.04)	-0.20 (-0.46,0.06)	-0.26 (-0.91,0.40)	-0.06 (-0.35,0.24)	-0.02 (-0.85,0.82)	-0.03 (-0.40,0.33)	**LC**		
**-0.32 (-0.61,-0.02)**	**-0.20 (-0.38,-0.02)**	-0.26 (-0.90,0.38)	-0.06 (-0.31,0.20)	-0.02 (-0.84,0.80)	-0.04 (-0.35,0.28)	-0.00 (-0.20,0.19)	**LF**	
**-0.63 (-0.94,-0.32)**	**-0.51 (-0.73,-0.30)**	-0.57 (-1.22,0.07)	**-0.37 (-0.63,-0.12)**	-0.33 (-1.15,0.49)	-0.35 (-0.72,0.02)	**-0.32 (-0.58,-0.05)**	**-0.32 (-0.50,-0.13)**	**CON**

Supplemental Table S6. League table: Long-term studies: ≥12 months. The values above the diet classes correspond to the difference in mean (95% CI) in

HbA1c (%) between the row and columns (i.e., the mean difference in average HbA1c between moderate-carbohydrate and Control diet is -0.63%)

Note. LC = low-carbohydrate, MC = moderate-carbohydrate, LF = low-fat, HP = high-protein, MD = Mediterranean, VEG = Vegetarian/Vegan, LGI = low GI/GL, RECOM = recommended, CON = control.

**KD**								
-0.93 (-2.15,0.29)	**MD**							
-1.00 (-2.06,0.06)	-0.07 (-0.67,0.53)	**LGI**						
-1.13 (-2.47,0.20)	-0.21 (-1.14,0.73)	-0.13 (-0.95,0.68)	**LC**					
-1.33 (-2.80,0.15)	-0.40 (-1.53,0.74)	-0.33 (-1.36,0.71)	-0.19 (-0.83,0.45)	**VEG**				
**-1.43 (-2.66,-0.20)**	-0.50 (-1.28,0.28)	-0.43 (-1.05,0.20)	-0.29 (-1.07,0.48)	-0.10 (-1.12,0.91)	**HP**			
**-1.53 (-2.69,-0.38)**	-0.61 (-1.27,0.06)	-0.53 (-1.00,-0.07)	-0.40 (-1.06,0.26)	-0.21 (-1.13,0.72)	-0.11 (-0.51,0.30)	**LF**		
**-1.77 (-3.14,-0.39)**	-0.84 (-1.83,0.16)	-0.77 (-1.64,0.10)	**-0.63 (-0.96,-0.31)**	-0.44 (-0.99,0.11)	-0.34 (-1.19,0.51)	-0.23 (-0.97,0.51)	**RECOM**	
**-1.89 (-3.01,-0.77)**	**-0.96 (-1.44,-0.48)**	**-0.89 (-1.24,-0.53)**	-0.75 (-1.56,0.05)	-0.56 (-1.59,0.47)	-0.46 (-1.07,0.15)	-0.35 (-0.81,0.10)	-0.12 (-0.99,0.75)	**CON**

Supplemental Table S7. League table: Short-term studies: ＜12 months. The values above the diet classes correspond to the difference in mean (95% CI) in
HbA1c (%) between the row and columns (i.e., the mean difference in average HbA1c between ketogenic and control diet is -1.89%)

Note. LC = low-carbohydrate, KD = ketogenic, LF = low-fat, HP = high-protein, MD = Mediterranean, VEG = Vegetarian/Vegan, LGI = low GI/GL, RECOM = recommended, CON = control.

**MC**							
-0.21 (-0.52,0.11)	**MD**						
-0.28 (-0.61,0.05)	-0.08 (-0.30,0.15)	**RECOM**					
-0.15 (-2.16,1.85)	0.05 (-1.96,2.06)	0.13 (-1.89,2.14)	**LGI**				
-0.48 (-0.96,0.00)	-0.27 (-0.67,0.12)	-0.20 (-0.64,0.24)	-0.33 (-2.36,1.71)	**HP**			
**-0.48 (-0.80,-0.16)**	**-0.27 (-0.43,-0.12)**	-0.20 (-0.45,0.06)	-0.33 (-2.33,1.68)	0.00 (-0.36,0.36)	**LF**		
**-0.56 (-0.94,-0.18)**	**-0.36 (-0.61,-0.10)**	-0.28 (-0.61,0.05)	-0.41 (-2.43,1.61)	-0.08 (-0.49,0.33)	-0.08 (-0.28,0.12)	**LC**	
**-0.81 (-1.12,-0.49)**	**-0.60 (-0.79,-0.41)**	**-0.53 (-0.76,-0.29)**	-0.65 (-2.66,1.36)	-0.33 (-0.74,0.08)	**-0.33 (-0.52,-0.14)**	-0.25 (-0.53,0.04)	**CON**

Supplemental Table S8. League table: Studies with sample size : ≥100. The values above the diet classes correspond to the difference in mean (95% CI) in
HbA1c (%) between the row and columns (i.e., the mean difference in average HbA1c between moderate-carbohydrate and control diet is -0.81%)

Note. LC = low-carbohydrate, MC = moderate-carbohydrate, LF = low-fat, HP = high-protein, MD = Mediterranean, LGI = low GI/GL, RECOM = recommended, CON = control.

**KD**								
-1.00 (-2.08,0.08)	**LGI**							
-1.02 (-2.24,0.20)	-0.02 (-0.58,0.55)	**LC**						
-1.11 (-2.41,0.19)	-0.11 (-0.84,0.61)	-0.10 (-0.66,0.46)	**VEG**					
**-1.42 (-2.62,-0.21)**	-0.42 (-0.94,0.11)	-0.40 (-0.98,0.18)	-0.30 (-1.07,0.47)	**HP**				
**-1.44 (-2.67,-0.21)**	-0.44 (-1.02,0.15)	-0.42 (-0.78,-0.06)	-0.32 (-0.75,0.10)	-0.02 (-0.66,0.62)	**RECOM**			
**-1.51 (-2.67,-0.36)**	-0.51 (-0.92,-0.10)	-0.50 (-0.97,-0.02)	-0.40 (-1.09,0.29)	-0.10 (-0.43,0.23)	-0.07 (-0.62,0.47)	**LF**		
**-1.63 (-2.94,-0.31)**	-0.63 (-1.38,0.12)	-0.61 (-1.41,0.18)	-0.52 (-1.45,0.42)	-0.21 (-0.93,0.50)	-0.19 (-1.03,0.64)	-0.12 (-0.76,0.52)	**MC**	
**-1.79 (-2.93,-0.65)**	**-0.79 (-1.14,-0.44)**	**-0.77 (-1.34,-0.21)**	-0.68 (-1.41,0.06)	-0.38 (-0.87,0.12)	-0.35 (-0.95,0.25)	-0.28 (-0.64,0.09)	-0.16 (-0.88,0.56)	**CON**

Supplemental Table S9. League table: Studies with sample size : ＜100. The values above the diet classes correspond to the difference in mean (95% CI) in
HbA1c (%) between the row and columns (i.e., the mean difference in average HbA1c between ketogenic and control diet is -1.79%)

Note. LC = low-carbohydrate, MC = moderate-carbohydrate, KD = ketogenic, LF = low-fat, HP = high-protein, VEG = Vegetarian/Vegan, LGI = low GI/GL, RECOM = recommended, CON = control.

**LC**							
-0.14 (-0.73,0.44)	**MC**						
-0.15 (-0.75,0.46)	-0.00 (-0.67,0.67)	**LGI**					
-0.18 (-0.65,0.30)	-0.03 (-0.44,0.38)	-0.03 (-0.61,0.55)	**MD**				
-0.24 (-0.88,0.40)	-0.10 (-0.70,0.50)	-0.10 (-0.83,0.63)	-0.07 (-0.55,0.41)	**HP**			
**-0.50 (-0.95,-0.05)**	-0.36 (-0.75,0.03)	-0.36 (-0.93,0.21)	-0.33 (-0.47,-0.18)	-0.26 (-0.72,0.20)	**LF**		
**-0.60 (-0.83,-0.37)**	-0.45 (-1.07,0.17)	-0.45 (-1.06,0.16)	-0.42 (-0.93,0.08)	-0.35 (-1.02,0.31)	-0.09 (-0.58,0.39)	**RECOM**	
**-0.89 (-1.37,-0.42)**	**-0.75 (-1.13,-0.36)**	**-0.75 (-1.31,-0.19)**	**-0.72 (-0.93,-0.50)**	**-0.65 (-1.15,-0.15)**	**-0.39 (-0.58,-0.20)**	-0.30 (-0.81,0.22)	**CON**

Supplemental Table S10. League table: Studies with drop-out rates : ≤10%. The values above the diet classes correspond to the difference in mean (95% CI) in
HbA1c (%) between the row and columns (i.e., the mean difference in average HbA1c between low-carbohydrate and control diet is -0.89%)

Note. LC = low-carbohydrate, MC = moderate-carbohydrate, LF = low-fat, HP = high-protein, MD = Mediterranean, LGI = low GI/GL, RECOM = recommended, CON = control.

**KD**										
-1.00 (-2.04,0.04)	**LGI**									
-1.18 (-2.48,0.13)	-0.18 (-0.96,0.61)	**VEG**								
-1.23 (-2.46,0.01)	-0.23 (-0.89,0.44)	-0.05 (-0.60,0.49)	**MC**							
**-1.35 (-2.62,-0.07)**	-0.35 (-1.08,0.39)	-0.17 (-0.69,0.35)	-0.12 (-0.54,0.30)	**MD**						
**-1.41 (-2.54,-0.28)**	-0.41 (-0.86,0.04)	-0.24 (-0.89,0.42)	-0.18 (-0.69,0.32)	-0.06 (-0.66,0.53)	**LF**					
**-1.41 (-2.57,-0.25)**	-0.41 (-0.92,0.10)	-0.24 (-0.93,0.46)	-0.18 (-0.75,0.38)	-0.06 (-0.71,0.58)	-0.00 (-0.24,0.24)	**HP**				
**-1.50 (-2.75,-0.25)**	-0.50 (-1.19,0.19)	-0.32 (-0.70,0.05)	-0.27 (-0.67,0.13)	-0.15 (-0.52,0.22)	-0.09 (-0.63,0.45)	-0.09 (-0.68,0.50)	**RECOM**			
**-1.52 (-2.67,-0.38)**	**-0.52 (-1.00,-0.04)**	-0.35 (-1.00,0.30)	-0.30 (-0.81,0.22)	-0.18 (-0.77,0.42)	-0.11 (-0.29,0.07)	-0.11 (-0.41,0.19)	-0.02 (-0.56,0.51)	**LC**		
**-1.73 (-2.85,-0.60)**	**-0.73 (-1.17,-0.28)**	-0.55 (-1.32,0.22)	-0.50 (-1.15,0.15)	-0.38 (-1.10,0.34)	-0.31 (-0.73,0.10)	-0.31 (-0.79,0.17)	-0.22 (-0.90,0.45)	-0.20 (-0.65,0.25)	**CON**	

Supplemental Table S11. League table: Studies with drop-out rates : ＞10%. The values above the diet classes correspond to the difference in mean (95% CI) in
HbA1c (%) between the row and columns (i.e., the mean difference in average HbA1c between ketogenic and control diet is -1.73%)

Note. LC = low-carbohydrate, MC = moderate-carbohydrate, KD = ketogenic, LF = low-fat, HP = high-protein, MD = Mediterranean, VEG = Vegetarian/Vegan, LGI = low GI/GL, RECOM = recommended, CON = control.

**KD**									
-1.00 (-2.05,0.05)	**LGI**								
-1.15 (-2.49,0.19)	-0.15 (-0.98,0.68)	**MD**							
-1.30 (-2.64,0.05)	-0.30 (-1.14,0.55)	-0.15 (-0.81,0.52)	**VEG**						
**-1.38 (-2.72,-0.04)**	-0.38 (-1.21,0.45)	-0.23 (-0.88,0.42)	-0.08 (-0.74,0.58)	**MC**					
**-1.62 (-2.91,-0.33)**	-0.62 (-1.37,0.13)	-0.47 (-1.01,0.07)	-0.32 (-0.71,0.06)	-0.24 (-0.78,0.29)	**RECOM**				
**-1.67 (-3.04,-0.31)**	-0.67 (-1.55,0.20)	-0.52 (-1.23,0.18)	-0.38 (-1.09,0.34)	-0.29 (-0.82,0.23)	-0.05 (-0.65,0.55)	**HP**			
**-1.78 (-3.15,-0.42)**	-0.78 (-1.65,0.09)	-0.63 (-1.33,0.07)	-0.49 (-1.19,0.22)	-0.40 (-0.93,0.12)	-0.16 (-0.75,0.43)	-0.11 (-0.45,0.23)	**LC**		
**-1.74 (-3.09,-0.40)**	-0.74 (-1.58,0.10)	-0.59 (-1.25,0.07)	-0.45 (-1.11,0.22)	-0.36 (-0.83,0.10)	-0.12 (-0.67,0.42)	-0.07 (-0.32,0.18)	0.04 (-0.20,0.28)	**LF**	
**-0.73 (-1.19,-0.27)**	-0.37 (-0.84,0.10)	-0.46 (-1.28,0.35)	-0.47 (-3.10,2.15)	-0.33 (-0.85,0.20)	-0.44 (-0.97,0.10)	0.06 (-0.46,0.57)	**-0.70 (-1.37,-0.02)**	**-2.11 (-3.37,-0.85)**	**CON**

Supplemental Table S12. League table: Studies without high risk of bias studies. The values above the diet classes correspond to the difference in mean (95% CI) in
HbA1c (%) between the row and columns (i.e., the mean difference in average HbA1c between ketogenic and control diet is -0.73%)

Note. LC = low-carbohydrate, MC = moderate-carbohydrate, KD = ketogenic, LF = low-fat, HP = high-protein, MD = Mediterranean, VEG = Vegetarian/Vegan, LGI = low GI/GL, RECOM = recommended, CON = control.

**MC**							
-0.39 (-1.06,0.27)	**MD**						
-0.56 (-1.19,0.08)	-0.16 (-0.60,0.27)	**LF**					
-0.63 (-1.59,0.33)	-0.24 (-1.08,0.60)	-0.08 (-0.78,0.63)	**HP**				
-0.78 (-1.63,0.07)	-0.39 (-1.24,0.46)	-0.22 (-1.03,0.58)	-0.15 (-1.22,0.92)	**LC**			
-0.88 (-2.53,0.77)	-0.49 (-2.13,1.16)	-0.32 (-1.97,1.32)	-0.25 (-2.04,1.55)	-0.10 (-1.71,1.51)	**VEG**		
**-1.18 (-2.00,-0.36)**	-0.79 (-1.60,0.02)	-0.63 (-1.44,0.19)	-0.55 (-1.64,0.54)	-0.40 (-1.14,0.33)	-0.30 (-1.73,1.13)	**RECOM**	
**-1.35 (-2.13,-0.57)**	**-0.96 (-1.67,-0.25)**	**-0.80 (-1.38,-0.21)**	-0.72 (-1.64,0.20)	-0.57 (-1.54,0.40)	-0.47 (-2.20,1.26)	-0.17 (-1.14,0.80)	**CON**

Supplemental Table S13. League table: Long-term studies: ≥12 months. The values above the diet classes correspond to the difference in mean (95% CI) in fasting glucose (mmol/L) between the row and columns (i.e., the mean difference in average fasting glucose between moderate-carbohydrate and control diet is -1.35mmol/L)

Note. LC = low-carbohydrate, MC = moderate-carbohydrate, LF = low-fat, HP = high-protein, MD = Mediterranean, VEG = Vegetarian/Vegan, RECOM = recommended, CON = control.

**VEG**									
-0.24 (-2.52,2.04)	**LC**								
-0.44 (-1.91,1.03)	-0.20 (-1.94,1.54)	**RECOM**							
-0.88 (-4.83,3.07)	-0.64 (-3.86,2.59)	-0.44 (-4.10,3.23)	**KD**						
-1.02 (-4.24,2.20)	-0.78 (-3.05,1.49)	-0.58 (-3.44,2.28)	-0.14 (-3.00,2.72)	**HP**					
-1.09 (-4.44,2.26)	-0.85 (-3.31,1.60)	-0.65 (-3.66,2.36)	-0.21 (-2.31,1.88)	-0.07 (-2.02,1.87)	**LGI**				
-1.44 (-5.27,2.39)	-1.20 (-4.28,1.88)	-1.00 (-4.54,2.54)	-0.56 (-3.36,2.24)	-0.42 (-3.11,2.27)	-0.35 (-2.21,1.51)	**MD**			
-1.56 (-5.40,2.28)	-1.32 (-4.41,1.76)	-1.12 (-4.67,2.42)	-0.68 (-3.49,2.12)	-0.54 (-3.25,2.16)	-0.47 (-2.34,1.40)	-0.12 (-1.98,1.74)	**MC**		
-1.46 (-4.44,1.52)	-1.22 (-3.14,0.70)	-1.02 (-3.61,1.57)	-0.58 (-3.17,2.01)	-0.44 (-1.65,0.77)	-0.37 (-1.89,1.16)	-0.02 (-2.42,2.39)	0.10 (-2.31,2.52)	**LF**	
-2.46 (-6.07,1.14)	-2.22 (-5.01,0.57)	-2.02 (-5.31,1.26)	-1.58 (-4.06,0.89)	-1.44 (-3.80,0.91)	**-1.37 (-2.70,-0.04)**	-1.02 (-2.33,0.28)	-0.90 (-2.22,0.42)	-1.00 (-3.03,1.02)	**CON**

Supplemental Table S14. League table: Short-term studies: ＜12 months. The values above the diet classes correspond to the difference in mean (95% CI) in fasting glucose (mmol/L) between the row and columns (i.e., the mean difference in average fasting glucose between low GI/GL and control diet is -1.37mmol/L)

Note. LC = low-carbohydrate, MC = moderate-carbohydrate, KD = ketogenic, LF = low-fat, HP = high-protein, MD = Mediterranean, VEG = Vegetarian/Vegan, LGI = low GI/GL, RECOM = recommended, CON = control.

**MC**						
-0.52 (-1.26,0.22)	**MD**					
-0.72 (-1.48,0.03)	-0.20 (-0.65,0.25)	**LF**				
-1.02 (-2.27,0.22)	-0.50 (-1.59,0.58)	-0.30 (-1.29,0.69)	**HP**			
-1.08 (-2.20,0.05)	-0.56 (-1.64,0.53)	-0.35 (-1.37,0.66)	-0.05 (-1.47,1.36)	**LC**		
**-1.08 (-2.08,-0.08)**	-0.56 (-1.55,0.43)	-0.36 (-1.41,0.69)	-0.06 (-1.50,1.38)	-0.00 (-1.40,1.39)	**RECOM**	
**-1.58 (-2.43,-0.74)**	**-1.06 (-1.74,-0.38)**	**-0.86 (-1.48,-0.24)**	-0.56 (-1.73,0.61)	-0.51 (-1.67,0.66)	-0.50 (-1.64,0.64)	**CON**

Supplemental Table S15. League table: Studies with sample size: ≥100. The values above the diet classes correspond to the difference in mean (95% CI) in fasting glucose (mmol/L) between the row and columns (i.e., the mean difference in average fasting glucose between moderate-carbohydrate and control diet is -1.58mmol/L)

Note. LC = low-carbohydrate, MC = moderate-carbohydrate, LF = low-fat, HP = high-protein, MD = Mediterranean, RECOM = recommended, CON = control.

**LC**								
-0.10 (-1.14,0.94)	**VEG**							
-0.42 (-3.17,2.34)	-0.32 (-3.26,2.63)	**KD**						
-0.63 (-2.60,1.34)	-0.53 (-2.76,1.70)	-0.21 (-2.14,1.71)	**LGI**					
-0.47 (-1.07,0.13)	-0.37 (-1.22,0.48)	-0.05 (-2.87,2.77)	0.16 (-1.89,2.22)	**RECOM**				
-0.78 (-2.59,1.04)	-0.68 (-2.76,1.41)	-0.36 (-2.55,1.83)	-0.14 (-1.19,0.90)	-0.31 (-2.21,1.60)	**HP**			
-1.09 (-3.01,0.82)	-0.99 (-3.18,1.19)	-0.68 (-2.89,1.54)	-0.46 (-1.55,0.63)	-0.62 (-2.63,1.38)	-0.32 (-1.27,0.63)	**MC**		
-1.22 (-2.96,0.52)	-1.12 (-3.15,0.91)	-0.80 (-2.94,1.33)	-0.59 (-1.51,0.33)	-0.75 (-2.59,1.09)	-0.44 (-0.96,0.07)	-0.13 (-0.93,0.68)	**LF**	
-1.86 (-3.74,0.03)	-1.76 (-3.91,0.40)	-1.44 (-3.55,0.67)	**-1.22 (-2.09,-0.36)**	-1.39 (-3.36,0.59)	**-1.08 (-1.96,-0.21)**	-0.76 (-1.58,0.05)	-0.64 (-1.36,0.08)	**CON**

Supplemental Table S16. League table: Studies with sample size : ＜100. The values above the diet classes correspond to the difference in mean (95% CI) in fasting glucose (mmol/L) between the row and columns (i.e., the mean difference in average fasting glucose between low GI/GL and control diet is -1.22mmol/L)

Note. LC = low-carbohydrate, MC = moderate-carbohydrate, KD = ketogenic, LF = low-fat, HP = high-protein, VEG = Vegetarian/Vegan, LGI = low GI/GL, RECOM = recommended, CON = control.

**LC**						
-0.47 (-2.53,1.59)	**MC**					
-0.55 (-2.62,1.51)	-0.08 (-1.30,1.13)	**HP**				
-0.68 (-1.70,0.35)	-0.21 (-2.51,2.09)	-0.12 (-2.43,2.18)	**RECOM**			
-0.91 (-2.83,1.00)	-0.44 (-1.36,0.47)	-0.36 (-1.31,0.59)	-0.24 (-2.41,1.94)	**MD**		
-1.22 (-3.09,0.65)	-0.75 (-1.60,0.10)	-0.67 (-1.53,0.20)	-0.54 (-2.68,1.59)	-0.31 (-0.70,0.09)	**LF**	
-1.74 (-3.69,0.22)	**-1.27 (-2.05,-0.48)**	**-1.18 (-2.21,-0.16)**	-1.06 (-3.27,1.15)	**-0.82 (-1.46,-0.19)**	-0.52 (-1.07,0.04)	**CON**

Supplemental Table S17. League table: Studies with drop-out rates : ≤10%. The values above the diet classes correspond to the difference in mean (95% CI) in fasting glucose (mmol/L) between the row and columns (i.e., the mean difference in average fasting glucose between moderate-carbohydrate and control diet is -1.27mmol/L)

Note. LC = low-carbohydrate, MC = moderate-carbohydrate, LF = low-fat, HP = high-protein, MD = Mediterranean, RECOM = recommended, CON = control.

**LF**									
0.07 (-0.90,1.04)	**LGI**								
-0.05 (-0.81,0.72)	-0.12 (-1.35,1.12)	**MC**							
0.29 (-1.87,2.44)	0.21 (-1.71,2.14)	0.33 (-1.95,2.62)	**KD**						
-0.20 (-0.62,0.21)	-0.28 (-1.33,0.78)	-0.16 (-1.03,0.71)	-0.49 (-2.68,1.70)	**HP**					
**-0.77 (-1.47,-0.07)**	-0.84 (-2.04,0.36)	-0.72 (-1.43,-0.01)	-1.06 (-3.32,1.21)	-0.56 (-1.38,0.25)	**LC**				
-0.81 (-1.99,0.37)	-0.88 (-2.41,0.65)	-0.76 (-1.79,0.26)	-1.10 (-3.56,1.36)	-0.61 (-1.86,0.65)	-0.04 (-1.12,1.03)	**VEG**			
-0.80 (-1.70,0.10)	-0.87 (-2.19,0.45)	-0.75 (-1.33,-0.17)	-1.08 (-3.42,1.25)	-0.59 (-1.58,0.40)	-0.03 (-0.84,0.78)	0.01 (-1.02,1.05)	**MD**		
-1.18 (-2.00,-0.36)	-1.25 (-2.52,0.02)	-1.13 (-1.71,-0.56)	-1.47 (-3.77,0.84)	-0.98 (-1.90,-0.05)	-0.41 (-1.07,0.25)	-0.37 (-1.22,0.48)	-0.38 (-0.98,0.21)	**RECOM**	
**-1.49 (-2.37,-0.61)**	**-1.56 (-2.44,-0.68)**	**-1.44 (-2.61,-0.28)**	-1.78 (-3.89,0.34)	**-1.29 (-2.26,-0.31)**	-0.72 (-1.85,0.41)	-0.68 (-2.15,0.80)	-0.69 (-1.95,0.57)	-0.31 (-1.52,0.90)	**CON**

Supplemental Table S18. League table: Studies with drop-out rates : ＞10%. The values above the diet classes correspond to the difference in mean (95% CI) in fasting glucose (mmol/L) between the row and columns (i.e., the mean difference in average fasting glucose between low-fat and control diet is -1.49mmol/L)

Note. LC = low-carbohydrate, MC = moderate-carbohydrate, KD = ketogenic, LF = low-fat, HP = high-protein, MD = Mediterranean, VEG = Vegetarian/Vegan, LGI = low GI/GL, RECOM = recommended, CON = control.

**MC**									
-0.17 (-1.27,0.94)	**LC**								
-0.11 (-2.74,2.52)	0.05 (-2.72,2.83)	**KD**							
-0.37 (-1.01,0.26)	-0.21 (-1.28,0.87)	-0.26 (-2.86,2.34)	**MD**						
-0.33 (-1.94,1.29)	-0.16 (-2.00,1.68)	-0.21 (-2.29,1.86)	0.05 (-1.52,1.61)	**LGI**					
-0.48 (-1.32,0.36)	-0.31 (-1.53,0.90)	-0.37 (-2.99,2.26)	-0.11 (-0.84,0.63)	-0.15 (-1.76,1.46)	**HP**				
-0.49 (-1.83,0.85)	-0.33 (-1.61,0.96)	-0.38 (-3.27,2.51)	-0.12 (-1.44,1.21)	-0.16 (-2.17,1.85)	-0.01 (-1.47,1.45)	**VEG**			
**-0.69 (-1.30,-0.09)**	-0.53 (-1.60,0.54)	-0.58 (-3.14,1.98)	-0.32 (-0.77,0.13)	-0.37 (-1.87,1.13)	-0.21 (-0.80,0.37)	-0.20 (-1.54,1.13)	**LF**		
-0.86 (-1.74,0.02)	-0.69 (-1.49,0.10)	-0.75 (-3.45,1.96)	-0.49 (-1.34,0.37)	-0.53 (-2.27,1.20)	-0.38 (-1.43,0.67)	-0.37 (-1.38,0.64)	-0.17 (-1.04,0.71)	**RECOM**	
**-1.38 (-2.02,-0.73)**	**-1.21 (-2.36,-0.07)**	-1.27 (-3.87,1.34)	**-1.01 (-1.61,-0.40)**	-1.05 (-2.64,0.53)	**-0.90 (-1.68,-0.12)**	-0.89 (-2.27,0.50)	**-0.68 (-1.20,-0.17)**	-0.52 (-1.47,0.43)	**CON**

Supplemental Table S19. League table: Studies without high risk of bias studies. The values above the diet classes correspond to the difference in mean (95% CI) in fasting glucose (mmol/L) between the row and columns (i.e., the mean difference in average fasting glucose between moderate-carbohydrate and control diet is -1.38mmol/L)

Note. LC = low-carbohydrate, MC = moderate-carbohydrate, KD = ketogenic, LF = low-fat, HP = high-protein, MD = Mediterranean, VEG = Vegetarian/Vegan, LGI = low GI/GL, RECOM = recommended, CON = control.

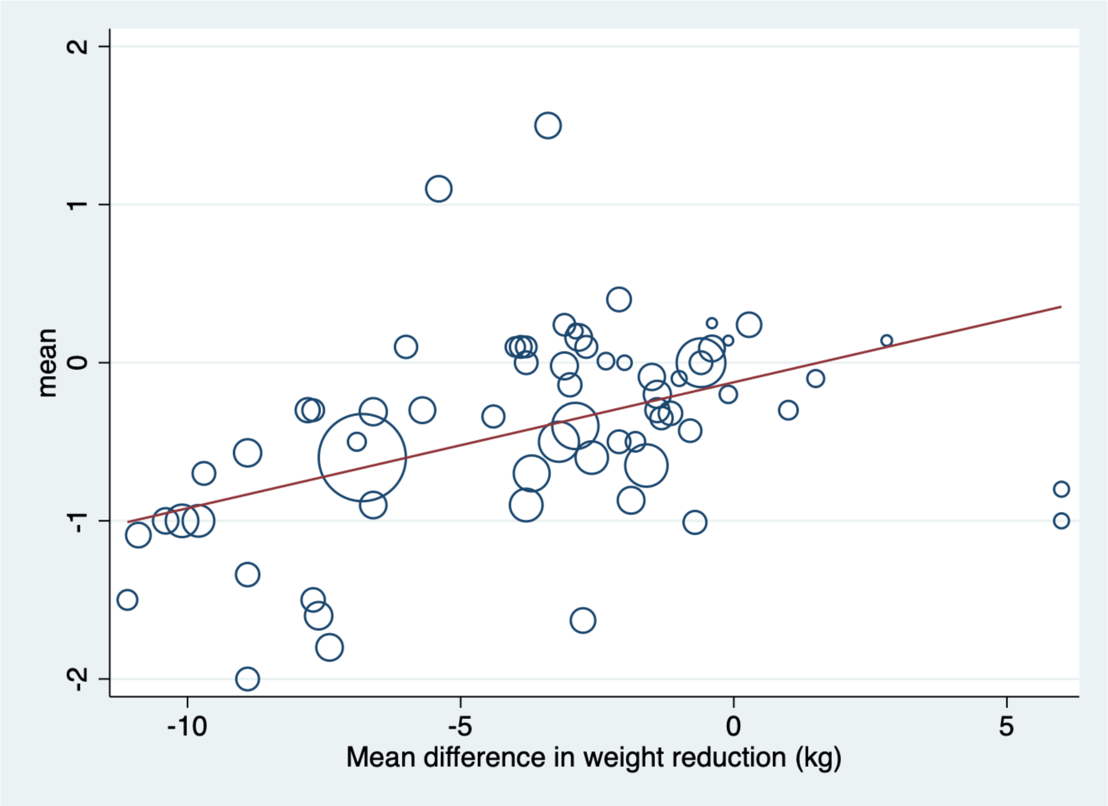

Supplemental Figure S5. Meta-regression showing the association between mean differences in HbA1c (%) and mean weight reduction of patients between dietary approaches

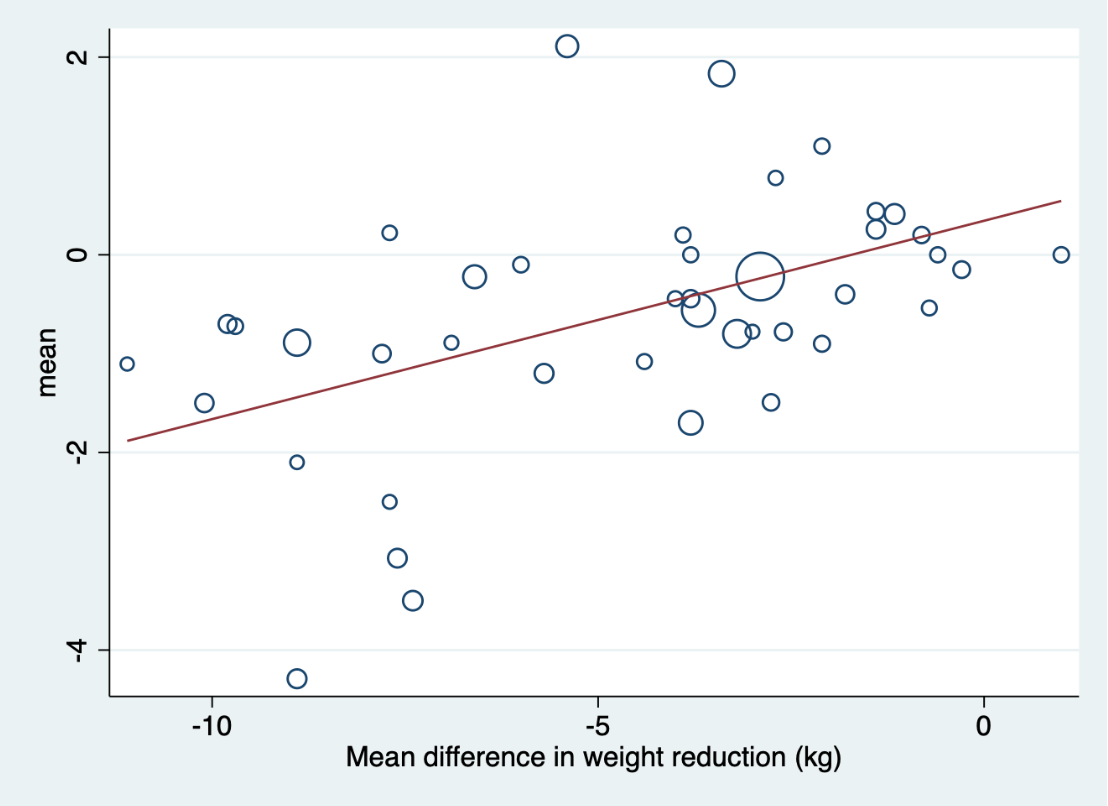

Supplemental Figure S6. Meta-regression showing the association between mean differences in fasting glucose (mmol/L) and mean weight reduction of patients between dietary approaches

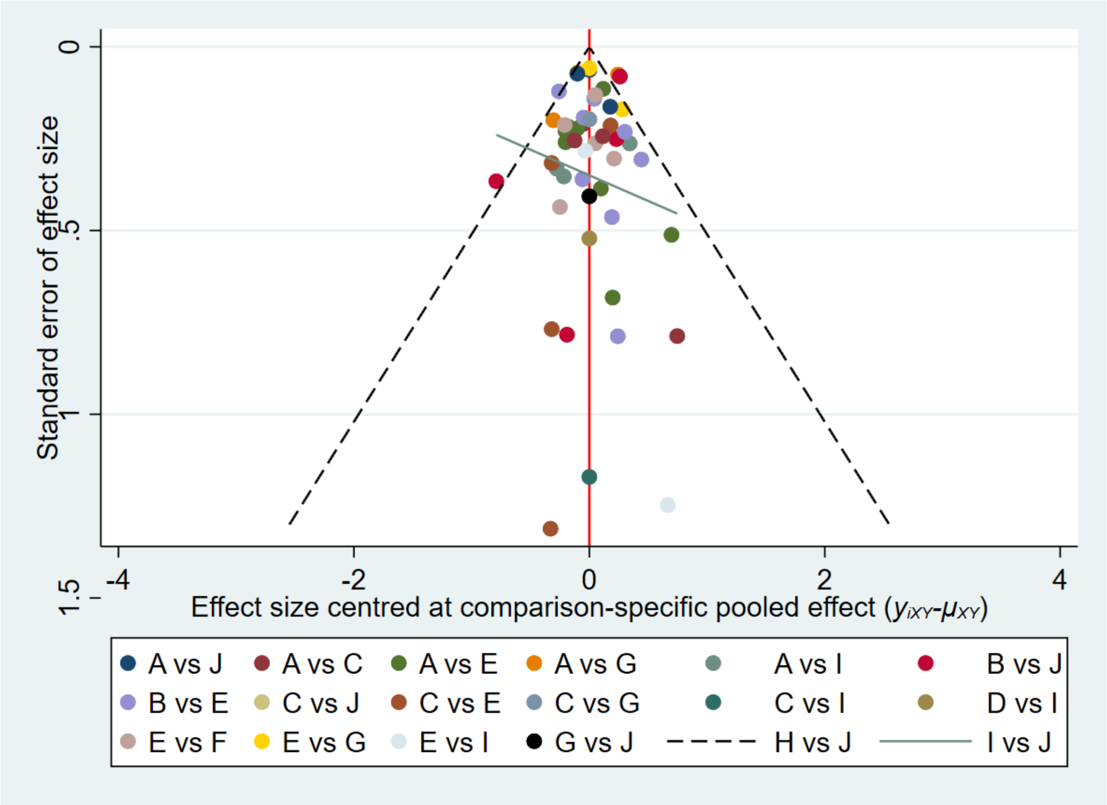

Supplemental Figure S7. Comparison-adjusted funnel plot for HbA1c involving all studies comparing all dietary approaches.

Note. A = control diet, B = low-carbohydrate diet, C = moderate-carbohydrate diet, D = ketogenic diet, E = low-fat diet, F = high-protein diet, G = Mediterranean diet, H = Vegetarian/Vegan diet, I = low GI/GL diet, J = recommended diet.

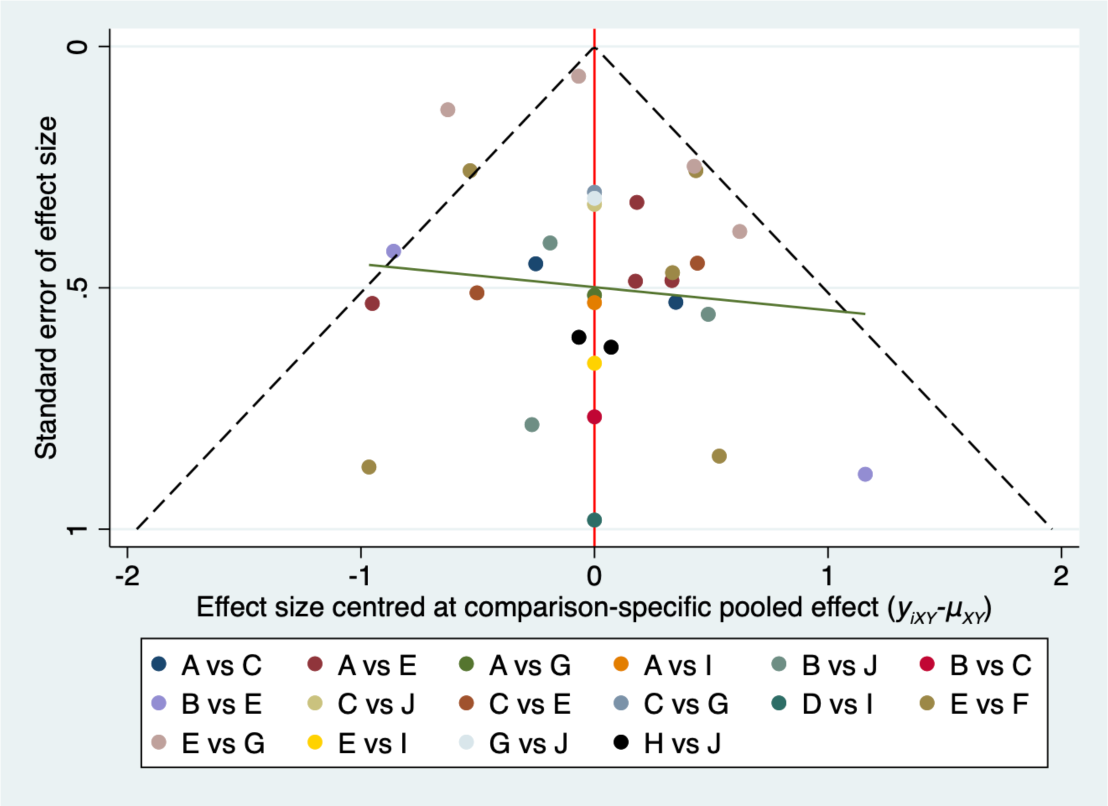

Supplemental Figure S8. Comparison-adjusted funnel plot for fasting glucose involving all studies comparing all dietary approaches.

Note. A = control diet, B = low-carbohydrate diet, C = moderate-carbohydrate diet, D = ketogenic diet, E = low-fat diet, F = high-protein diet, G = Mediterranean diet, H = Vegetarian/Vegan diet, I = low GI/GL diet, J = recommended diet.

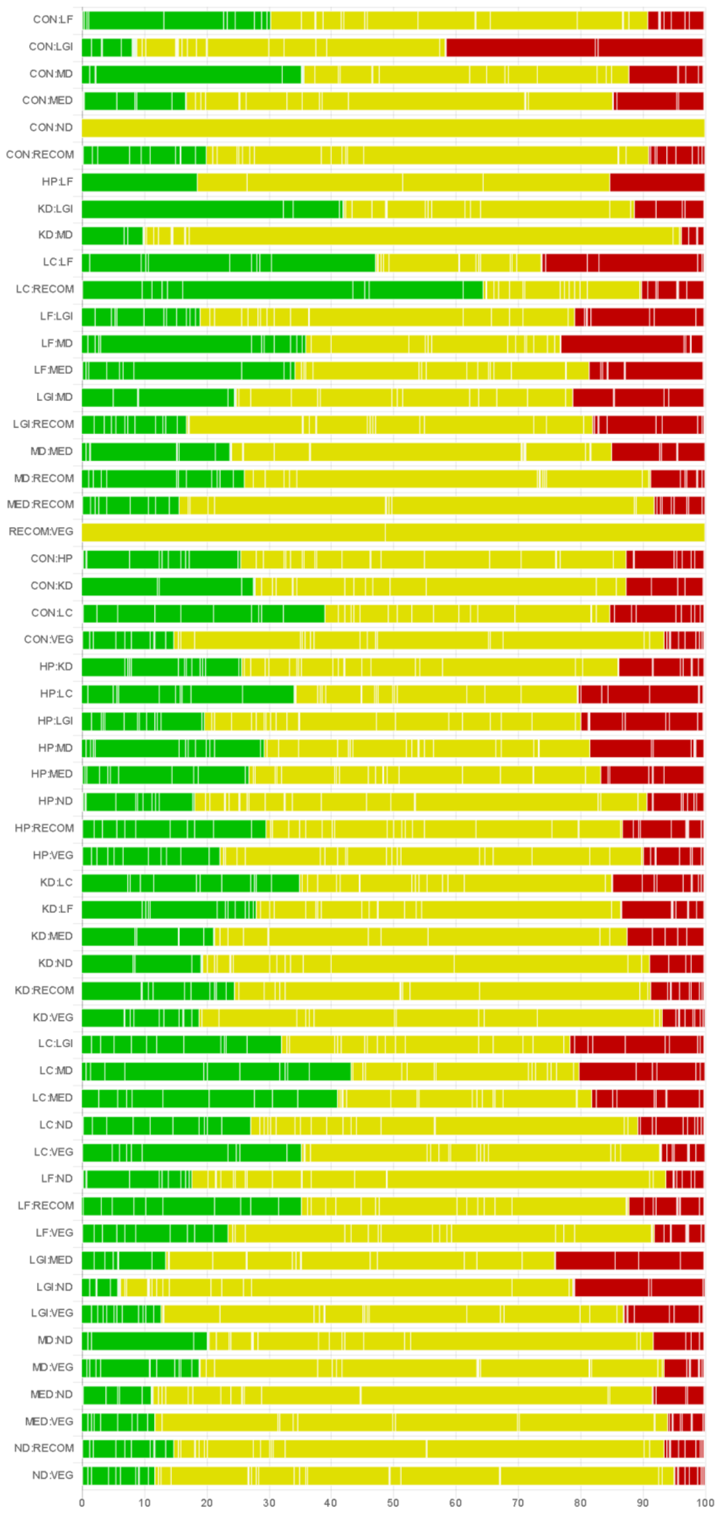

Supplemental Figure S9. Bar-graph showing for every comparison the percentage of information coming from low (green), moderate (yellow) and high (red) risk of bias studies for HbA1c

Note. CON = control, LC = low-carbohydrate, MC = moderate-carbohydrate, KD = ketogenic, LF = low-fat, HP = high-protein, MED = Mediterranean, ND=Nordic, VEG = Vegetarian/Vegan, LGI = low GI/GL, RECOM = recommended.

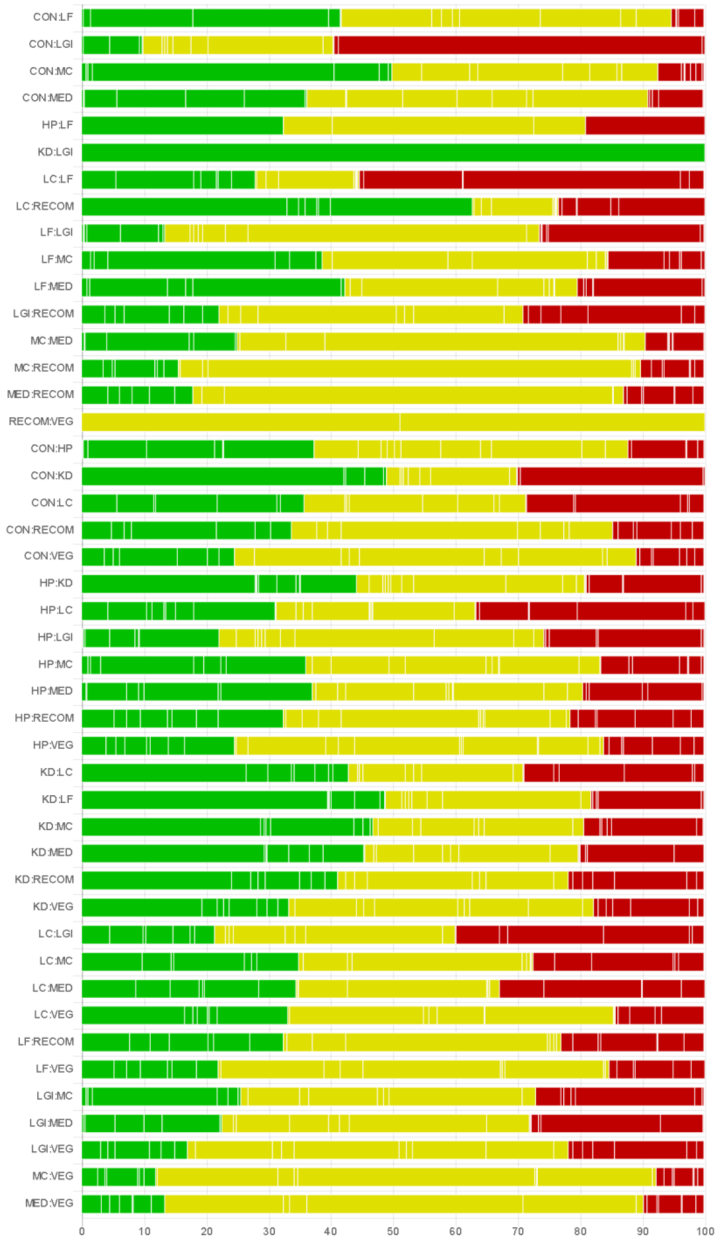

Supplemental Figure S10. Bar-graph showing for every comparison the percentage of information coming from low (green), moderate (yellow) and high (red) risk of bias studies for fasting glucose

Note. CON = control, LC = low-carbohydrate, MC = moderate-carbohydrate, KD = ketogenic, LF = low-fat, HP = high-protein, MED = Mediterranean, ND=Nordic, VEG = Vegetarian/Vegan, LGI = low GI/GL, RECOM = recommended.

Supplemental Table S20. GRADE evaluation for HbA1c and all comparisons between the different dietary approaches using CINeMA
Our judgements described below are based on the recommendations of the online documentation of CINeMA (https://cinema.ispm.unibe.ch).

Comparison	Number of studies	Within-study bias	Reporting bias	Indirectness	Imprecision	Heterogeneity	Incoherence	Confidence rating*
Mixed evidence								
CON:LF	8	Some concerns	Low risk	No concerns	No concerns	Some concerns	No concerns	Moderate
CON:LGI	3	Some concerns	Some concerns	No concerns	No concerns	No concerns	No concerns	Moderate
CON:MC	2	Some concerns	Low risk	No concerns	No concerns	Some concerns	No concerns	Moderate
CON:MC	3	Some concerns	Low risk	No concerns	No concerns	No concerns	No concerns	Moderate
CON:ND	1	Some concerns	Low risk	No concerns	Some concerns	No concerns	Major concerns	Very low
CON:RECOM	1	Some concerns	Low risk	No concerns	No concerns	Some concerns	No concerns	Moderate
HP:LF	6	Some concerns	Low risk	No concerns	No concerns	Major concerns	Major concerns	Very low
KD:LGI	1	Some concerns	Low risk	No concerns	Some concerns	Some concerns	No concerns	Low
KD:MC	1	Some concerns	Low risk	No concerns	Some concerns	No concerns	No concerns	Low
LC:LF	8	No concerns	Low risk	No concerns	No concerns	Major concerns	Some concerns	Low

LC:RECOM	4	No concerns	Low risk	No concerns	Some concerns	No concerns	Major concerns	Low
LF:LGI	2	Some concerns	Some concerns	No concerns	Some concerns	No concerns	No concerns	Moderate
LF:MC	5	Some concerns	Low risk	No concerns	Some concerns	Some concerns	No concerns	Low
LF:MD	4	Some concerns	Low risk	No concerns	Some concerns	No concerns	No concerns	Moderate
LGI:MC	1	Some concerns	Some concerns	No concerns	Some concerns	Some concerns	No concerns	Low
LGI:RECOM	1	Some concerns	Low risk	No concerns	Some concerns	No concerns	No concerns	Moderate
MC:MD	1	Some concerns	Low risk	No concerns	No concerns	Major concerns	No concerns	Low
MC:RECOM	1	Some concerns	Low risk	No concerns	Some concerns	No concerns	No concerns	Moderate
MD:RECOM	2	Some concerns	Low risk	No concerns	Some concerns	No concerns	No concerns	Moderate
RECOM:VEG	2	Some concerns	Low risk	No concerns	Some concerns	No concerns	Major concerns	Low
Indirect evidence								
CON:HP	0	Some concerns	Low risk	No concerns	No concerns	Some concerns	Major concerns	Low
CON:KD	0	Some concerns	Low risk	No concerns	No concerns	No concerns	Major concerns	Low

CON:LC	0	Some concerns	Low risk	No concerns	No concerns	Some concerns	Major concerns	Low
CON:VEG	0	Some concerns	Low risk	No concerns	No concerns	Some concerns	Major concerns	Low
HP:KD	0	Some concerns	Low risk	No concerns	Some concerns	No concerns	Major concerns	Low
HP:LC	0	Some concerns	Low risk	No concerns	No concerns	Major concerns	Major concerns	Very low
HP:LGI	0	Some concerns	Low risk	No concerns	Some concerns	Some concerns	Major concerns	Very low
HP:MC	0	Some concerns	Low risk	No concerns	Some concerns	Some concerns	Major concerns	Very low
HP:MD	0	Some concerns	Low risk	No concerns	Some concerns	Some concerns	Major concerns	Very low
HP:ND	0	Some concerns	Low risk	No concerns	Some concerns	No concerns	Major concerns	Low
HP:RECOM	0	Some concerns	Low risk	No concerns	Some concerns	Some concerns	Major concerns	Very low
HP:VEG	0	Some concerns	Low risk	No concerns	Major concerns	No concerns	Major concerns	Very low
KD:LC	0	Some concerns	Low risk	No concerns	Some concerns	No concerns	Major concerns	Low
KD:LF	0	Some concerns	Low risk	No concerns	No concerns	Some concerns	Major concerns	Low

KD:MD	0	Some concerns	Low risk	No concerns	Some concerns	No concerns	Major concerns	Low
KD:ND	0	Some concerns	Low risk	No concerns	No concerns	No concerns	Major concerns	Low
KD:RECOM	0	Some concerns	Low risk	No concerns	No concerns	Some concerns	Major concerns	Low
KD:VEG	0	Some concerns	Low risk	No concerns	Some concerns	Some concerns	Major concerns	Very low
LC:LGI	0	Some concerns	Low risk	No concerns	Some concerns	Some concerns	Major concerns	Very low
LC:MC	0	No concerns	Low risk	No concerns	Some concerns	Some concerns	Major concerns	Low
LC:MD	0	No concerns	Low risk	No concerns	No concerns	Major concerns	Major concerns	Very low
LC:ND	0	Some concerns	Low risk	No concerns	Some concerns	No concerns	Major concerns	Low
LC:VEG	0	Some concerns	Low risk	No concerns	Major concerns	No concerns	Major concerns	Very low
LF:ND	0	Some concerns	Low risk	No concerns	Some concerns	No concerns	Major concerns	Low
LF:RECOM	0	Some concerns	Low risk	No concerns	No concerns	Major concerns	Major concerns	Very low
LF:VEG	0	Some concerns	Low risk	No concerns	Some concerns	Some concerns	Major concerns	Very low

LGI:MD	0	Some concerns	Low risk	No concerns	Some concerns	Some concerns	Major concerns	Very low
LGI:ND	0	Some concerns	Low risk	No concerns	No concerns	Some concerns	Major concerns	Low
LGI:VEG	0	Some concerns	Low risk	No concerns	Major concerns	No concerns	Major concerns	Very low
MC:ND	0	Some concerns	Low risk	No concerns	Some concerns	No concerns	Major concerns	Low
MC:VEG	0	Some concerns	Low risk	No concerns	Major concerns	No concerns	Major concerns	Very low
MD:ND	0	Some concerns	Low risk	No concerns	No concerns	Some concerns	Major concerns	Low
MD:VEG	0	Some concerns	Low risk	No concerns	Major concerns	No concerns	Major concerns	Very low
ND:RECOM	0	Some concerns	Low risk	No concerns	Some concerns	Some concerns	Major concerns	Very low
ND:VEG	0	Some concerns	Low risk	No concerns	Some concerns	No concerns	Major concerns	Low

*The overall confidence rating was determined according to the following criteria: all five domains with "no concerns": high confidence; one or two domains with
"some concerns": downgrading by one level; three domains with "some concerns": downgrading by two levels; at least one domain with "major concerns":
downgrading by two levels

Note. CON = control, LC = low-carbohydrate, MC = moderate-carbohydrate, KD = ketogenic, LF = low-fat, HP = high-protein, MD = Mediterranean, ND=Nordic, VEG = Vegetarian/Vegan, LGI = low GI/GL, RECOM = recommended.

Supplemental Table S21. GRADE evaluation for fasting glucose and all comparisons between the different dietary approaches using CINeMA

Comparison	Number of studies	Within-study bias	Reporting bias	Indirectness	Imprecision	Heterogeneity	Incoherence	Confidence rating
Mixed evidence								
CON:LF	5	Some concerns	Low risk	No concerns	No concerns	Some concerns	No concerns	Moderate
CON:LGI	1	Major concerns	Low risk	No concerns	No concerns	Some concerns	No concerns	Low
CON:MC	1	No concerns	Low risk	No concerns	No concerns	No concerns	No concerns	High
CON:MD	1	Some concerns	Low risk	No concerns	No concerns	No concerns	No concerns	Moderate
HP:LF	5	Some concerns	Low risk	No concerns	No concerns	Major concerns	No concerns	Low
KD:LGI	1	No concerns	Low risk	No concerns	Major concerns	No concerns	No concerns	Low
LC:LF	3	Major concerns	Low risk	No concerns	Some concerns	Some concerns	No concerns	Low
LC:RECOM	3	No concerns	Low risk	No concerns	Some concerns	No concerns	No concerns	Moderate
LF:LGI	1	Some concerns	Low risk	No concerns	Some concerns	Some concerns	No concerns	Low
LF:MC	3	Some concerns	Low risk	No concerns	Some concerns	No concerns	No concerns	Moderate

LF:MD	4	No concerns	Low risk	No concerns	No concerns	Some concerns	No concerns	Moderate
LGI:RECOM	1	Some concerns	Low risk	No concerns	Some concerns	No concerns	No concerns	Moderate
MC:MD	1	Some concerns	Low risk	No concerns	Some concerns	No concerns	No concerns	Moderate
MC:RECOM	1	Some concerns	Low risk	No concerns	No concerns	Some concerns	No concerns	Moderate
MD:RECOM	1	Some concerns	Low risk	No concerns	Some concerns	No concerns	No concerns	Moderate
RECOM:VEG	2	Some concerns	Low risk	No concerns	Some concerns	Some concerns	No concerns	Low
Indirect evidence								
CON:HP	0	Some concerns	Low risk	No concerns	No concerns	Some concerns	No concerns	Moderate
CON:KD	0	No concerns	Low risk	No concerns	Major concerns	No concerns	No concerns	Low
CON:LC	0	No concerns	Low risk	No concerns	Some concerns	No concerns	No concerns	Moderate
CON:RECOM	0	Some concerns	Low risk	No concerns	Some concerns	Some concerns	No concerns	Low
CON:VEG	0	Some concerns	Low risk	No concerns	Some concerns	Some concerns	No concerns	Low
HP:KD	0	No concerns	Low risk	No concerns	Major concerns	No concerns	No concerns	Low

HP:LC	0	Major concerns	Low risk	No concerns	Some concerns	Some concerns	No concerns	Low
HP:LGI	0	Some concerns	Low risk	No concerns	Major concerns	No concerns	No concerns	Low
HP:MC	0	Some concerns	Low risk	No concerns	Some concerns	Some concerns	No concerns	Low
HP:MD	0	Some concerns	Low risk	No concerns	No concerns	Major concerns	No concerns	Low
HP:RECOM	0	Some concerns	Low risk	No concerns	Some concerns	No concerns	No concerns	Moderate
HP:VEG	0	Some concerns	Low risk	No concerns	Major concerns	No concerns	No concerns	Low
KD:LC	0	No concerns	Low risk	No concerns	Major concerns	No concerns	No concerns	Low
KD:LF	0	No concerns	Low risk	No concerns	Major concerns	No concerns	No concerns	Low
KD:MC	0	No concerns	Low risk	No concerns	Major concerns	No concerns	No concerns	Low
KD:MD	0	No concerns	Low risk	No concerns	Major concerns	No concerns	No concerns	Low
KD:RECOM	0	No concerns	Low risk	No concerns	Major concerns	No concerns	No concerns	Low
KD:VEG	0	Some concerns	Low risk	No concerns	Major concerns	No concerns	No concerns	Low

LC:LGI	0	Major concerns	Low risk	No concerns	Some concerns	Some concerns	No concerns	Low
LC:MC	0	Some concerns	Low risk	No concerns	Some concerns	No concerns	No concerns	Moderate
LC:MD	0	No concerns	Low risk	No concerns	Some concerns	Some concerns	No concerns	Moderate
LC:VEG	0	Some concerns	Low risk	No concerns	Major concerns	No concerns	No concerns	Low
LF:RECOM	0	Some concerns	Low risk	No concerns	Some concerns	No concerns	No concerns	Moderate
LF:VEG	0	Some concerns	Low risk	No concerns	Major concerns	No concerns	No concerns	Low
LGI:MC	0	Some concerns	Low risk	No concerns	Major concerns	No concerns	No concerns	Low
LGI:MD	0	Some concerns	Low risk	No concerns	Major concerns	No concerns	No concerns	Low
LGI:VEG	0	Some concerns	Some concerns	No concerns	Major concerns	No concerns	No concerns	Low
MC:VEG	0	Some concerns	Low risk	No concerns	Some concerns	Some concerns	No concerns	Low
MD:VEG	0	Some concerns	Low risk	No concerns	Major concerns	No concerns	No concerns	Low

*The overall confidence rating was determined according to the following criteria: all five domains with “no concerns”: high confidence; one or two domains with

“some concerns”: downgrading by one level; three domains with “some concerns”: downgrading by two levels; at least one domain with “major concerns”:
downgrading by two levels.

Note. CON = control, LC = low-carbohydrate, MC = moderate-carbohydrate, KD = ketogenic, LF = low-fat, HP = high-protein, MD = Mediterranean, VEG = Vegetarian/Vegan, LGI = low GI/GL, RECOM = recommended.

Weight change after 12 months (kg)									
**KD**	NA	NA	NA	NA	NA	NA	NA	NA	NA
-2.85 (-14.01,8.31)	**MD**	0.84 (-1.69,3.36)	-1.94 (-5.18,1.29)	-0.39 (-3.25,2.47)	-0.61 (-0.95,-0.28)	-0.88 (-1.42,-0.34)	-2.69 (-8.14,2.76)	**-1.79 (-3.41,-0.17)**	**-3.37 (-4.84,-1.89)**
-1.91 (-13.28,9.46)	0.94 (-1.55,3.44)	**MC**	-2.78 (-6.86,1.30)	-1.23 (-4.93,2.47)	-1.45 (-3.97,1.06)	-1.72 (-4.26,0.82)	-3.53 (-9.07,2.02)	-2.63 (-5.48,0.22)	**-3.20 (-6.97,-1.44)**
-3.30 (-14.67,8.08)	-0.44 (-2.68,1.79)	-1.39 (-4.71,1.94)	**HP**	1.55 (-2.74,5.85)	1.33 (-1.89,4.55)	1.06 (-2.18,4.31)	-0.75 (-7.07,5.58)	0.15 (-3.44,3.74)	-1.42 (-4.95,2.10)
-3.26 (-14.73,8.22)	-0.40 (-3.11,2.31)	-1.35 (-4.95,2.26)	0.04 (-3.44,3.53)	**VEG**	-0.22 (-3.07,2.62)	-0.49 (-3.32,2.33)	-2.30 (-8.35,3.75)	-1.40 (-3.76,0.96)	-2.98 (-6.16,0.21)
-3.47 (-14.62,7.69)	**-0.61 (-0.95,-0.28)**	-1.56 (-4.04,0.93)	-0.17 (-2.38,2.04)	-0.21 (-2.90,2.48)	**LF**	-0.27 (-0.69,0.15)	-2.08 (-7.52,3.37)	-1.18 (-2.77,0.42)	**-2.75 (-4.19,-1.32)**
-3.73 (-14.89,7.43)	**-0.88 (-1.41,-0.34)**	-1.82 (-4.33,0.69)	-0.43 (-2.68,1.82)	-0.47 (-3.14,2.19)	-0.26 (-0.68,0.16)	**LC**	-1.81 (-7.26,3.64)	-0.91 (-2.46,0.64)	**-2.49 (-3.98,-0.99)**
-4.20 (-15.19,6.79)	-1.35 (-3.30,0.61)	-2.29 (-5.22,0.64)	-0.90 (-3.84,2.03)	-0.94 (-4.24,2.35)	-0.73 (-2.66,1.19)	-0.47 (-2.44,1.50)	**LGI**	0.90 (-4.67,6.47)	-0.68 (-6.28,4.93)
-4.66 (-15.88,3.57)	**-1.80 (-3.14,-0.47)**	**-2.75 (-5.47,-0.03)**	-1.36 (-3.92,1.21)	-1.40 (-3.76,0.96)	-1.19 (-2.49,0.11)	-0.93 (-2.17,0.32)	-0.46 (-2.75,1.84)	**RECOM**	-1.58 (-3.71,0.56)
**-6.26 (-17.34,-0.82)**	**-3.41 (-4.82,-1.99)**	**-3.35 (-7.01,-1.69)**	**-2.96 (-5.57,-0.36)**	**-3.00 (-6.02,-0.01)**	**-2.79 (-4.17,-1.42)**	**-2.53 (-3.97,-1.10)**	**-2.06 (-3.49,-0.63)**	-1.60 (-3.48,0.27)	**CON**
Weight change after 6 months (kg)									

Supplemental Table S22. League table: Mean differences in body weight at 6 and 12 months intervention. The values above the diet classes correspond to the difference in mean (95% CI) in fasting glucose (mmol/L) between the row and columns (i.e., the mean difference in weight change between ketogenic and control diet is -6.26kg)

Note. LC = low-carbohydrate, MC = moderate-carbohydrate, KD = ketogenic, LF = low-fat, HP = high-protein, MD = Mediterranean, VEG = Vegetarian/Vegan, LGI = low GI/GL, RECOM = recommended, CON = control.

Supplemental Table S23. SUCRA ranking for the dietary approaches on weight change

	Weight change after 6 months (kg)	SUCRA (%)	Weight change after 12 months (kg)	SUCRA (%)
1	Ketogenic	74.6	Mediterranean	81.9
2	Mediterranean	74.3	Moderate-carbohydrate	77.7
3	Moderate-carbohydrate	72.5	Vegetarian/Vegan	65.1
4	High-protein	57.4	Low-fat	62.0
5	Vegetarian/Vegan	57.1	Low-carbohydrate	49.0
6	Low-fat	54.3	High-protein	34.2
7	Low-carbohydrate	41.8	Recommended	30.8
8	Low GI/GL	34.7	Low GI/GL	30.4
9	Recommended	21.0	Control	8.9
10	Control	2.4	—	—

Supplementary References of excluded studies

1. de Bont, A.J.; Baker, I.A.; St Leger, A.S.; Sweetnam, P.M.; Wragg, K.G.; Stephens, S.M.; Hayes, T.M. A randomised controlled trial of the effect of low fat diet advice on dietary response in insulin independent diabetic women. *Diabetologia* **1981**, *21*, 529–533, https://doi.org/10.1007/bf00281543.
2. Milne, R.M.; Mann, J.I.; Chisholm, A.W.; Williams, S.M. Long-term comparison of three dietary prescriptions in the treatment of NIDDM. *Diabetes Care* **1994**, *17*, 74–80, https://doi.org/10.2337/diacare.17.1.74.
3. Nielsen, J.V.; Jönsson, E.; Nilsson, A.K. Lasting improvement of hyperglycaemia and bodyweight: low-carbohydrate diet in type 2 diabetes. A brief report. *Ups. J. Med. Sci.* **2005**, *110*, 179–183, https://doi.org/10.3109/2000-1967-182.
4. Fraser, A.; Abel, R.; Lawlor, D.A.; Fraser, D.; Elhayany, A. A modified Mediterranean diet is associated with the greatest reduction in alanine aminotransferase levels in obese type 2 diabetes patients: results of a quasi-randomised controlled trial. *Diabetologia* **2008**, *51*, 1616–1622, https://doi.org/10.1007/s00125-008-1049-1.
5. Wolever, T.M.S.; Mehling, C.; Chiasson, J.L.; Josse, R.G.; Leiter, L.A.; Maheux, P.; Rabasa-Lhoret, R.; Rodger, N.W.; Ryan, E.A. Low glycaemic index diet and disposition index in type 2 diabetes (the Canadian trial of Carbohydrates in Diabetes): a randomised controlled trial. *Diabetologia* **2008**, *51*, 1607–1615, https://doi.org/10.1007/s00125-008-1093-x.
6. Haimoto, H.; Iwata, M.; Wakai, K.; Umegaki, H. Long-term effects of a diet loosely restricting carbohydrates on HbA1c levels, BMI and tapering of sulfonylureas in type 2 diabetes: a 2-year follow-up study. *Diabetes Res. Clin. Pract.* **2008**, *79*, 350–356, https://doi.org/10.1016/j.diabres.2007.09.009.
7. Jenkins, D.J.; Kendall, C.W.; McKeown-Eyssen, G.; Josse, R.G.; Silverberg, J.; Booth, G.L.; Vidgen, E.; Josse, A.R.; Nguyen, T.H.; Corrigan, S.; et al. Effect of a low-glycemic index or a high-cereal fiber diet on type 2 diabetes: a randomized trial. *JAMA* **2008**, *300*, 2742–2753, https://doi.org/10.1001/jama.2008.808.
8. Haimoto, H.; Sasakabe, T.; Wakai, K.; Umegaki, H. Effects of a low-carbohydrate diet on glycemic control in outpatients with severe type 2 diabetes. *Nutr. Metab.* **2009**, *6*, 1–5, https://doi.org/10.1186/1743-7075-6-21.
9. Moses, R.G.; Barker, M.; Winter, M.; Petocz, P.; Brand-Miller, J.C. Can a low-glycemic index diet reduce the need for insulin in gestational diabetes mellitus? A randomized trial. *Diabetes Care* **2009**, *32*, 996–1000, https://doi.org/10.2337/dc09-0007.
10. Turner-McGrievy, G.M.; Barnard, N.; Jenkins, D.; Cohen, J.; Gloede, L.; Green, A. *Relation of the Glycemic Index with Body Weight and Glycemic Control Among Participants with Type 2 Diabetes Following a Low-Fat Vegan Diet or a Conventional Diabetes Diet for 22 Weeks*; the 27th Annual Scientific Meeting of the Obesity-Society, Washington, DC, 2009; p. S131.
11. Kahleova, H.; Matoulek, M.; Malinska, H.; Oliyarnik, O.; Kazdova, L.; Neskudla, T.; Skoch, A.; Hajek, M.; Hill, M.; Kahle, M.; et al. Vegetarian diet improves insulin resistance and oxidative stress markers more than conventional diet in subjects with Type 2 diabetes. *Diabet. Med.* **2011**, *28*, 549–559, https://doi.org/10.1111/j.1464-5491.2010.03209.x.

12. Cao, A.; Sun, L.; Cui, J. Effects of A Low - Carbohydrate Diet and A Low - Fat Diet on Weight and Glycemic Control in Type 2 Diabetics Mellitus. *Chin. Gen. Prac.* **2011**, *14*, 52–53,56, https://doi.org/10.3969/j.issn.1007-9572.2011.01.016.
13. Larsen, R.N.; Mann, N.J.; Maclean, E.; Shaw, J.E. The effect of high-protein, low-carbohydrate diets in the treatment of type 2 diabetes: A 12 month randomised controlled trial. *Diabetologia* **2011**, *54*, 731–740, https://doi.org/10.1007/s00125-010-2027-y.
14. Shyam, S.; Arshad, F.; Nisak, M.Y.B.; Safie, N.S.; Kamaruddin, N.A.; Abdul Ghani, R.; Abdul Wahab, N.A. *Effect of low gi diet vs low fat diet on metabolic risk markers in women post gestational diabetes mellitus (PGDM): A preliminary finding*; the 4th International Congress on Prediabetes and the Metabolic Syndrome, Madrid, Spain, 2011; p. 87.
15. Krebs, J.D.; Elley, C.R.; Parry-Strong, A.; Lunt, H.; Drury, P.L.; Bell, D.A.; Robinson, E.; Moyes, S.A.; Mann, J. *Two Year Randomised Controlled Trial of High-Protein Versus High-Carbohydrate Diet in Type 2 Diabetes: Diabetes Excess Weight Loss (DEWL)*; the 71st Scientific Sessions of the American Diabetes Association, San Diego, CA, USA, 2011; p. A213.
16. Tobias, D.K.; Hu, F.B.; Chavarro, J.; Rosner, B.; Mozaffarian, D.; Zhang, C. Healthful dietary patterns and type 2 diabetes mellitus risk among women with a history of gestational diabetes mellitus. *Arch. Gerontol. Geriatr.* **2012**, *172*, 1566–1572, https://doi.org/10.1001/archinternmed.2012.3747.
17. Davis, N.J.; Tomuta, N.; Isasi, C.R.; Leung, V.; Wylie-Rosett, J. Diabetes-specific quality of life after a low-carbohydrate and low-fat dietary intervention. *Diabetes Educ.* **2012**, *38*, 250–255, https://doi.org/10.1177/0145721711436132.
18. Jesudason, D.R.; Pedersen, E.; Clifton, P.M. Weight-loss diets in people with type 2 diabetes and renal disease: a randomized controlled trial of the effect of different dietary protein amounts. *Am. J. Clin. Nutr.* **2013**, *98*, 494–501, https://doi.org/10.3945/ajcn.113.060889.
19. Shyam, S.; Arshad, F.; Abdul Ghani, R.; Wahab, N.A.; Safii, N.S.; Nisak, M.Y.B.; Chinna, K.; Kamaruddin, N.A. Low glycaemic index diets improve glucose tolerance and body weight in women with previous history of gestational diabetes: A six months randomized trial. *Nutr. J.* **2013**, *12*, 68, https://doi.org/10.1186/1475-2891-12-68.
20. Fernemark, H.; Jaredsson, C.; Bunjaku, B.; Rosenqvist, U.; Nystrom, F.H.; Guldbrand, H. A randomized cross-over trial of the postprandial effects of three different diets in patients with type 2 diabetes. *PLoS One* **2013**, *8*, e79324, https://doi.org/10.1371/journal.pone.0079324.
21. Shyam, S.; Arshad, F.; Ghani, R.A.; Wahab, N.A.; Mohd Yusof, B.N.; Safii, N.S.; Chinna, K.; Kamaruddin, N.A. Low glycaemic index diet aids management of fasting blood sugar and body weight in asian women with previous history of gestational diabetes mellitus. *J. Diabetes* **2013**, *5*, 27, https://doi.org/10.1111/1753-0407.12032.
22. Mayer, S.B.; Jeffreys, A.S.; Olsen, M.K.; McDuffie, J.R.; Feinglos, M.N.; Yancy, W.S., Jr. Two diets with different haemoglobin A1c and antiglycaemic medication effects despite similar weight loss in type 2 diabetes. *Diabetes Obes. Metab.* **2014**, *16*, 90–93, https://doi.org/10.1111/dom.12191.

23. Tay, J.; Luscombe-Marsh, N.D.; Thompson, C.H.; Noakes, M.; Buckley, J.D.; Wittert, G.A.; Yancy, W.S., Jr.; Brinkworth, G.D. A Very Low-Carbohydrate, Low-Saturated Fat Diet for Type 2 Diabetes Management: A Randomized Trial. *Diabetes Care* **2015**, *37*, 2909–2918, https://doi.org/10.2337/dc14-0845.
24. Watson, N.A.; Dyer, K.A.; Buckley, J.D.; Brinkworth, G.D.; Coates, A.M.; Parfitt, G.; Howe, P.R.C.; Noakes, M.; Dye, L.; Chadwick, H.; et al. A randomised trial comparing low-fat diets differing in carbohydrate and protein ratio, combined with regular moderate intensity exercise, on glycaemic control, cardiometabolic risk factors, food cravings, cognitive function and psychological wellbeing in adults with type 2 diabetes: Study protocol. *Contemp. Clin. Trials* **2015**, *45*, 217–225, https://doi.org/10.1016/j.cct.2015.11.001.
25. Louie, J.C.; Markovic, T.P.; Ross, G.P.; Foote, D.; Brand-Miller, J.C. Effect of a low glycaemic index diet in gestational diabetes mellitus on post-natal outcomes after 3 months of birth: a pilot follow-up study. *Matern. Child. Nutr.* **2015**, *11*, 409–414, https://doi.org/10.1111/mcn.12039.
26. Tay, J.; Zajac, I.T.; Thompson, C.H.; Luscombe-Marsh, N.D.; Danthiir, V.; Noakes, M.; Buckley, J.D.; Wittert, G.A.; Brinkworth, G.D. A randomised-controlled trial of the effects of very low-carbohydrate and high-carbohydrate diets on cognitive performance in patients with type 2 diabetes. *Br. J. Nutr.* **2016**, 23, 1–9, https://doi.org/10.1017/s0007114516004001.
27. Stentz, F.B.; Brewer, A.; Wan, J.; Garber, C.; Daniels, B.; Sands, C.; Kitabchi, A.E. Remission of pre-diabetes to normal glucose tolerance in obese adults with high protein versus high carbohydrate diet: randomized control trial. *BMJ Open Diabetes Res. Care* **2016**, *4*, e000258, https://doi.org/10.1136/bmjdrc-2016-000258.
28. Wolever, T.M.; Chiasson, J.L.; Josse, R.G.; Leiter, L.A.; Maheux, P.; Rabasa-Lhoret, R.; Rodger, N.W.; Ryan, E.A. Effects of Changing the Amount and Source of Dietary Carbohydrates on Symptoms and Dietary Satisfaction Over a 1-Year Period in Subjects with Type 2 Diabetes: Canadian Trial of Carbohydrates in Diabetes (CCD). *Can. J. Diabetes* **2017**, *41*, 164–176, https://doi.org/10.1016/j.jcjd.2016.08.223.
29. Kahleova, H.; Klementova, M.; Herynek, V.; Skoch, A.; Herynek, S.; Hill, M.; Mari, A.; Pelikanova, T. The Effect of a Vegetarian vs Conventional Hypocaloric Diabetic Diet on Thigh Adipose Tissue Distribution in Subjects with Type 2 Diabetes: A Randomized Study. *J. Am. Coll. Nutr.* **2017**, *36*, 364–369, https://doi.org/10.1080/07315724.2017.1302367.
30. Crowley, M.J.; Edelman, D.; Voils, C.I.; Maciejewski, M.L.; Coffman, C.J.; Jeffreys, A.S.; Turner, M.J.; Gaillard, L.A.; Hinton, T.A.; Strawbridge, E.; et al. Jump starting shared medical appointments for diabetes with weight management: Rationale and design of a randomized controlled trial. *Contemp. Clin. Trials* **2017**, *58*, 1–12, https://doi.org/10.1016/j.cct.2017.04.004.
31. Marco Benedi, M.V.; Perez Calahorra, S.; Bea Sanz, A.M.; Baila Rueda, L.; Lamiquiz Moneo, I.; Cenarro, A.; Civeira, F.; Mateo Gallego, R. A Randomized, Open-Label Study to Investigate the Effect of a High Protein Diet Compared to a Normoprotein Diet on Hydrocarbon Metabolism in Patients with Diabetes or Prediabetes and

Obesity. *Atherosclerosis* **2017**, *263*, E263, https://doi.org/10.1016/j.atherosclerosis.2017.06.851.
32. Beck-Nielsen, H. A Reduced-carbohydrate Diet High in Monounsaturated Fats in Type 2 Diabetes **2017**. Available online: https://clinicaltrials.gov/show/NCT03068078. (accessed on 1 August 2022)
33. Sato, J.; Kanazawa, A.; Makita, S.; Hatae, C.; Komiya, K.; Shimizu, T.; Ikeda, F.; Tamura, Y.; Ogihara, T.; Mita, T.; et al. A randomized controlled trial of 130 g/day low-carbohydrate diet in type 2 diabetes with poor glycemic control. *Clin. Nutr.* **2017**, *36*, 992–1000, https://doi.org/10.1016/j.clnu.2016.07.003.
34. Asle Mohammadi Zadeh, M.; Kargarfard, M.; Marandi, S.M.; Habibi, A. Diets along with interval training regimes improves inflammatory & anti-inflammatory condition in obesity with type 2 diabetes subjects. *J. Diabetes Metab. Disord.* **2018**, *17*, 253–267, https://doi.org/10.1007/s40200-018-0368-0.
35. Tay, J.; Thompson, C.H.; Luscombe-Marsh, N.D.; Wycherley, T.P.; Noakes, M.; Buckley, J.D.; Wittert, G.A.; Yancy, W.S.; Brinkworth, G.D. Effects of an energy-restricted low-carbohydrate, high unsaturated fat/low saturated fat diet versus a high-carbohydrate, low-fat diet in type 2 diabetes: A 2-year randomized clinical trial. *Diabetes Obes. Metab.* **2018**, *20*, 858–871, https://doi.org/10.1111/dom.13164.
36. Torres-Peña, J.D.; Garcia-Rios, A.; Delgado-Casado, N.; Gomez-Luna, P.; Alcala-Diaz, J.F.; Yubero-Serrano, E.M.; Gomez-Delgado, F.; Leon-Acuña, A.; Lopez-Moreno, J.; Camargo, A.; et al. Mediterranean diet improves endothelial function in patients with diabetes and prediabetes: a report from the CORDIOPREV study. *Atherosclerosis* **2018**, *269*, 50–56, https://doi.org/10.1016/j.atherosclerosis.2017.12.012.
37. Björklund, A. Comparison of Low, Moderate and High Carbohydrate Diet on Insulin Requirements and Metabolic Control in Type 1 Diabetes **2018**. Available online: https://clinicaltrials.gov/show/NCT03761186. (accessed on 1 August 2022)
38. Mattei, J.; Bigornia, S.J.; Sotos-Prieto, M.; Scott, T.; Gao, X.; Tucker, K.L. The mediterranean diet and two-year changes in cognitive function in puerto rican adults with vs. Without Type 2 Diabetes. *Diabetes* **2018**, *67(Supplement_1)*, 192–LB, https://doi.org/10.2337/db18-192-LB.
39. Crowder, C.M.; Jelley, D.; Condren, M.; Chalmers, L.; Graef, J.L. The T1dlocho study: effects of a low-carbohydrate, nonketogenic diet vs. standard diabetes diet on glycemic control in young adults with type 1 diabetes. *Diabetes* **2019**, *68*, https://doi.org/10.2337/db19-72-LB.
40. Mason, A.E.; Saslow, L.R.; Moran, P.J.; Kim, S.; Abousleiman, H.; Richler, R.; Schleicher, S.; Goldman, V.M.; Hartman, A.; Leung, C.; et al. Lipid findings from the Diabetes Education to Lower Insulin, Sugars, and Hunger (DELISH) Study. *Nutr. Metab.* **2019**, *16*, 58, https://doi.org/10.1186/s12986-019-0383-2.
41. Stentz, F. Pathobiology of Remission of Type 2 Diabetes **2019**. Available online: https://clinicaltrials.gov/show/NCT03832725. (accessed on 1 August 2022)
42. Garbutt, J.D.W.; England, C.; Jones, A.G.; Andrews, R.C.; Johnson, L. Are changes in a low-carbohydrate, high-fat diet pattern associated with subsequent changes in HbA1c during an intensive diet and physical activity intervention? *Diabet. Med.* **2020**, *37*, 44, https://doi.org/10.1111/dme.14245.

43. Gram-Kampmann, E.M.; Hansen, C.D.; Hugger, M.B.; Jensen, J.M.; Brond, J.C.; Hermann, P.; Olsen, M.H.; Krag, A.; Beck-Nielsen, H.; Hojlund, K. Effects of a six-month low-carbohydrate diet in patients with type 2 diabetes on glycaemic control, body composition and cardiovascular risk factors. *Diabetologia* **2020**, *63*, S297, https://doi.org/10.1007/s00125-020-05221-5.
44. Tucker, S.; Stentz, F. Effect of macronutrients on metabolic parameters and remission of type 2 diabetes. *J. Investig. Med.* **2020**, *68*, 656–657, https://doi.org/10.1136/jim-2020-SRM.544.
45. Athinarayanan, S.J.; Hallberg, S.J.; McKenzie, A.L.; Lechner, K.; King, S.; McCarter, J.P.; Volek, J.S.; Phinney, S.D.; Krauss, R.M. Impact of a 2-year trial of nutritional ketosis on indices of cardiovascular disease risk in patients with type 2 diabetes. *Cardiovasc. Diabetol.* **2020**, *19*, 208, https://doi.org/10.1186/s12933-020-01178-2.
46. Blindbaek, S.L.; Moller, D.M.; Gram-Kampmann, E.M.; Olsen, M.H.; Hojlund, K.; Grauslund, J. Changes in Retinal Microvasculature Parameters after Low-Carbohydrate, High-Fat Diet in Type 2 Diabetes: A Randomized-Controlled Trial of Danish Type 2 Diabetic Patients. *Eur. J. Ophthalmol.* **2020**, *30*, 30, https://doi.org/10.1177/1120672120961310.
47. Kobayashi, M.; Miura, T.; Miura, K.; Hiroyama, N.; Akashi, K. Effect of a Moderate Carbohydrate-Restricted Diet on DPP-4 Inhibitor Action among Individuals with Type 2 Diabetes Mellitus: A 6-Month Intervention Study. *J. Nutr. Sci. Vitaminol.* **2020**, *66*, 114–118, https://doi.org/10.3177/jnsv.66.114.
48. Tay, J.; Thompson, C.H.; Luscombe-Marsh, N.D.; Noakes, M.; Buckley, J.D.; Wittert, G.A.; Brinkworth, G.D. Nutritional adequacy of very low- and high-carbohydrate, low saturated fat diets in adults with type 2 diabetes: A secondary analysis of a 2-year randomised controlled trial. *Diabetes Res. Clin. Pract.* **2020**, *170*, 108501, https://doi.org/10.1016/j.diabres.2020.108501.
49. Ren, M.; Zhang, H.; Qi, J.; Hu, A.; Jiang, Q.; Hou, Y.; Feng, Q.; Ojo, O.; Wang, X. An almond-based low carbohydrate diet improves depression and glycometabolism in patients with type 2 diabetes through modulating gut microbiota and glp-1: A randomized controlled trial. *Nutrients* **2020**, *12*, 1–21, https://doi.org/10.3390/nu12103036.
50. Lawson, D.; Stentz, F. Myonectin is a marker of remission of type 2 diabetes in obese human subjects. *J. Investig. Med.* **2021**, *69*, 516, https://doi.org/10.1136/jim-2021-SRMC.245.
51. Buso, M.E.C.; Seimon, R.V.; McClintock, S.; Muirhead, R.; Atkinson, F.S.; Brodie, S.; Dodds, J.; Zibellini, J.; Das, A.; Wild-Taylor, A.L.; et al. Can a Higher Protein/Low Glycemic Index vs. a Conventional Diet Attenuate Changes in Appetite and Gut Hormones Following Weight Loss? A 3-Year PREVIEW Sub-study. *Front. Nutr.* **2021**, *8*, 640538, https://doi.org/10.3389/fnut.2021.640538.
52. Dorans, K.S.; Bazzano, L.A.; Qi, L.; He, H.; Appel, L.J.; Samet, J.M.; Chen, J.; Mills, K.T.; Nguyen, B.T.; O'Brien, M.J.; et al. Low-carbohydrate dietary pattern on glycemic outcomes trial (ADEPT) among individuals with elevated hemoglobin A1c: study protocol for a randomized controlled trial. *Trials* **2021**, *22*, 108, https://doi.org/10.1186/s13063-020-05001-x.

53. Moriconi, E.; Camajani, E.; Fabbri, A.; Lenzi, A.; Caprio, M. Very-low-calorie ketogenic diet as a safe and valuable tool for long-term glycemic management in patients with obesity and type 2 diabetes. *Nutrients* **2021**, *13*, 1–15, https://doi.org/10.3390/nu13030758.
54. Han, Y.; Cheng, B.; Guo, Y.; Wang, Q.; Yang, N.; Lin, P. A Low-Carbohydrate Diet Realizes Medication Withdrawal: A Possible Opportunity for Effective Glycemic Control. *Front. Endocrinol.* **2021**, *12*, 779636, https://doi.org/10.3389/fendo.2021.779636.
55. Papamichou, D.; Panagiotakos, D.B.; Holmes, E.; Koutsakis, P.; Katsoulotos, H.; Loo, R.L.; Itsiopoulos, C. The rationale and design of a Mediterranean diet accompanied by time restricted feeding to optimise the management of type 2 diabetes: The MedDietFast randomised controlled trial. *Nutr. Metab. Cardiovasc. Dis.* **2022**, *32*, 220–230, https://doi.org/10.1016/j.numecd.2021.09.031.
